# Experimental–numerical analysis of added resistance to container ships under presence of wind–wave loads

**DOI:** 10.1371/journal.pone.0221453

**Published:** 2019-08-20

**Authors:** Wei Wang, Tiecheng Wu, Dagang Zhao, Chunyu Guo, Wanzhen Luo, Yongjie Pang

**Affiliations:** 1 College of Shipbuilding Engineering, Harbin Engineering University, Harbin, China; 2 School of Marine Engineering and Technology, Sun Yat-sen University, Zhuhai, China; University of Nottingham, UNITED KINGDOM

## Abstract

Experimental and numerical analyses performed on a scaled-down model of a 1900TEU container-ship are reported herein. Wind-tunnel and towing-tank experiments along with computational-fluid-dynamic simulations were performed to obtain (1) wind-load coefficients for superstructure of container ship at different wind angles under full-load operating conditions; (2) wave resistance of the model sans the superstructure under different wave conditions; and (3) combined wind–wave resistance of the model in the head waves coupled with a fluctuating wind. Wind-tunnel experiments were first performed to determine wind-load coefficients concerning of the superstructure at different wind angles. Subsequently, the obtained wind-load coefficients from the wind tunnel test were compared against numerical and empirically obtained results to validate the applicability of the applied numerical methods. Next, the wave-induced resistance to ship motion was investigated via a series of towing-tank experiments and numerical simulations to analyze the resistance and motion of ship under wavy conditions. Finally, characteristics of the added resistance to ship motion under conditions of combined wind–wave load were analyzed, and the coupling between ship motion and combined wind–wave load was used to investigate the changes in added resistance under different load scenarios. The results reveal that combined wind–wave load causes the resistance to ship motion to exceed the algebraic sum of the corresponding resistances under standalone wind- and wave-load conditions. The additional resistance was observed to be a combined manifestation of resistances induced by ship motion and wave-parameter alterations.

## 1. Introduction

Combined wind and wave loads induce complex effects on ship and the correspondence resistance to ship motion. Studies concerning wind resistances observed under isolated conditions tend to often neglect the effects of sea-surface roughness on wind-field gradients, whereas those concerning wave resistance alone neglect the effects of fluctuating wind fields, which exist under actual oceanic conditions, and wind–wave field couplings. Elucidation and evaluation of these additional resistances to ship motion under actual oceanic conditions require in-depth investigations to be performed concerning the effects of combined wind and wave loads.

Although most studies concerning wind and wave resistances of ships have exclusively considered the action of corresponding loads under isolated conditions, predicted values of the added resistance reported therein have been observed to be highly accurate. Regarding wind resistance, Isherwood [1] and Hong et al. [2] proposed an empirical formula for determining the wind-load coefficient based on regression of wind-tunnel test data. Kulkarni et al. [3] compared results obtained from numerical (computational fluid dynamics (CFD)) calculations against wind-tunnel test data to analyze detailed flow fields and corresponding wind-load coefficients at different wind angles of superstructures. Fujiwara et al. [4] performed wind-tunnel experiments to compare and analyze lateral forces and yaw moments acting on a container ship at different wind angles. Forrest et al. [5], Thornber [6], Jiang [7], and Mehta [8] performed large-eddy simulations (LES) to simulate the flow field around the ship superstructures numerically and analyze the detached eddies formed by the passage of wind through the superstructures.

Regarding studies concerning added resistance induced by the wave, Arribas [9] employed momentum- and radiated-energy-based techniques to theoretically calculate wave resistance of ships in the presence of regular and irregular waves, thereby observing that the radiated-energy method produces results highly consistent with experimental data. Seo et al. [10] performed OpenFOAM simulations to predict the resistance and motion of the KRISO container ship (KCS) under conditions involving waves of five different wavelengths. Their results demonstrated that large vortices mainly form around the stern, and that the wave resistance significantly increases at wavelengths of the order of the ship length. Paulsen et al. [11] developed a fully nonlinear domain-decomposed solver for calculating wave loads acting on structures in the time domain. Their proposed approach essentially coupled a nonlinear potential-flow method with a Navier–Stokes solver employing the volume-of-fluid technique to solve air-water two-phase flow problems. Afshary et al. [12] proposed a method for calculating wave resistance based on high-order finite-differences, with nolinear overset grids being used to solve linearized potential-flow equations. Chen et al. [13] proposed the use of an OpenFOAM-based numerical solver for simulating wave-induced rolling of two-dimensional rectangular barges. They reported that the observed viscous effect tends to dampen as well as enhance the magnitude of rolling motion. Duan [14] proposed a time-domain method for simulating the hydrodynamic performance of offshore structures in the presence of waves based on the body–boundary integral equations. This method employed the transient Green function, and an improved time-domain solution was obtained to prevent high-frequency oscillations of normal derivatives within the transient Green function. Bakhoday Paskyabi et al. [15] investigated the effects of boundary-layer interactions between waves and wind fields on the sea surface and performed a series of experiments to determine the effects of waves on air flows as well as those of air flows on wavy interfaces. Ma et al. [16] performed numerical simulations to investigate the combined effects of winds and waves on spar-type offshore floating wind turbines. Kalvig et al. [17] proposed a CFD-based method for simulating the effects of wave motion on wind fields and the consequent driving of waves.

Although studies concerning added resistance to ship motion caused by standalone wind or wave load have recently become highly detailed, limited discussion of the corresponding resistance observed under combined wind–wave actions has been included in the available literature. To address this gap, the authors in this study performed a series of wind-tunnel and towing-tank experiments along with CFD simulations, to study the generation mechanism of the additional resistance observed during motion of a 1900TEU container-ship model under presence of combined wind–wave loads. This study analyzes the relationship between ship motion and added resistance under the influence of several wave parameters and wind–wave couplings. Results provide insight into how combined wind–wave actions differ from isolated actions of wind and wave loads. Finally, the authors believe that findings of this study serve as a useful reference with regard to prediction of ship resistance, ship power, ship motion and flow field characteristics under wind-wave coupling.

The remainder of this paper is organized as follows. The methodology is described in detail in section 2, wherein section 2.1 reports the model geometries, section 2.2 covers the wind tunnel tests for determining wind-load coefficients, and section 2.3 describes the towing-tank experiment for measuring added resistance due to wave loads. The numerical modeling and CFD mesh setup are described in detail in section 3, wherein section 3.1 reports the governing equations, section 3.2 covers the turbulence model and free-surface treatment, and sections 3.3, 3.4, 3.5 and 3.6 describe the Numerical wave generation and wave damping, dynamic fluid–body interaction model for rotational and translational motion, respectively. The computational and experimental results are extensively analyzed and discussed in section 4, the study of wind loads is reported in section 4.1, the investigation of added resistance due to wave loads is analyzed in section 4.2, and sections 4.3 describe the investigation of added resistance due to combined wind-wave loads. Sections 5 and 6 provide a summary of this study and its limitations and recommendations for future research.

## 2. Methodology

### 2.1 Model geometries

**Fig 1:**
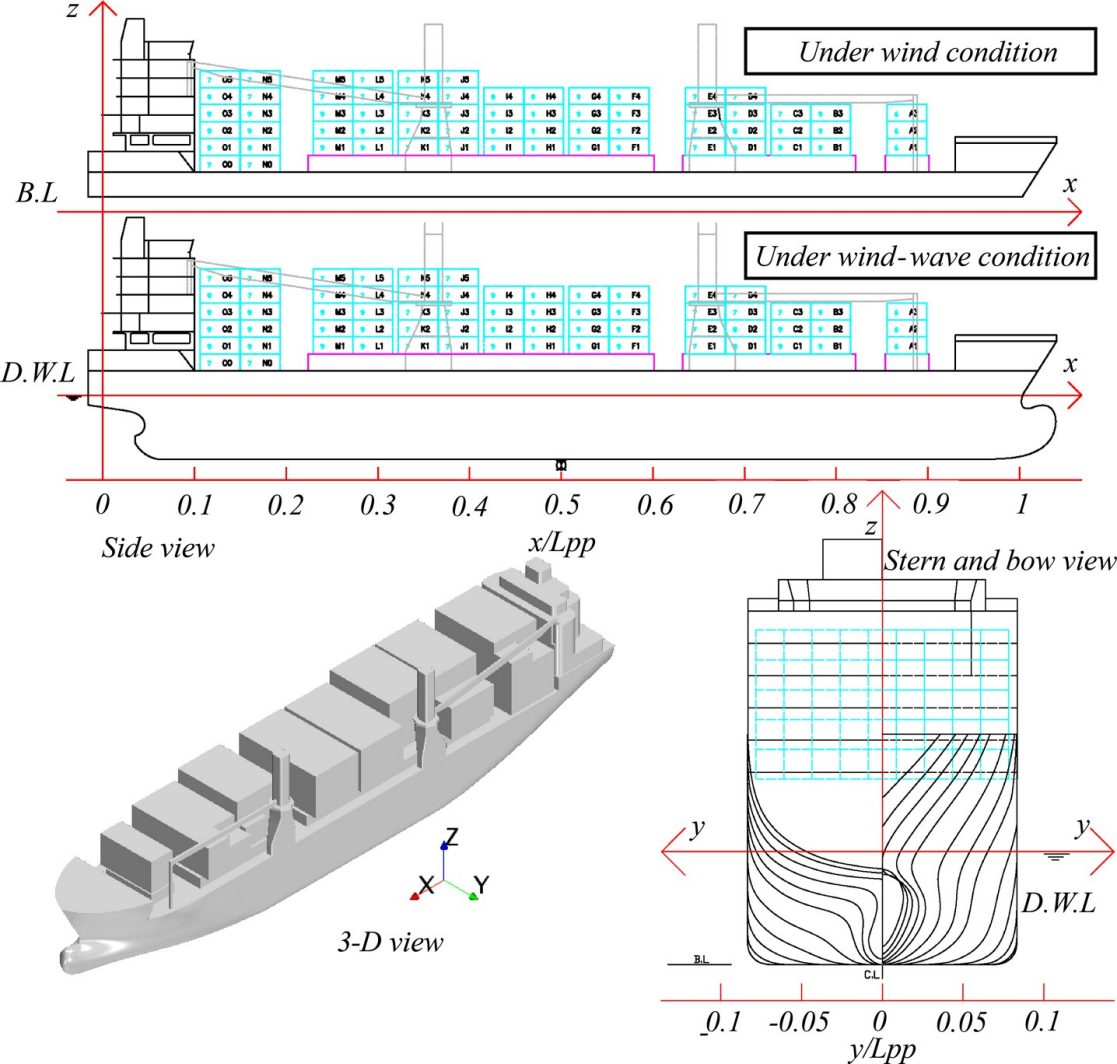
1900TEU ship model.

The test models used in this study were manufactured using fiberglass. The principal dimensions of models used have been listed in Table 1 with the model geometry being shown in Fig 1. The wind-load model includes parts located above the waterline with containers being fully loaded and no containers remaining within the crane-side area. The wave-load model is without superstructure. This model was equipped with turbulence stimulators (wires measuring 1.0 mm in diameter) positioned at 5% of the distance between perpendiculars located aft of the forward perpendicular.

**Table 1:** Parameters concerning 1900TEU ship model.

Main dimensions	Actual ship	Experimental model		CFD model	
		Wave condition	Wind condition	Wave or wind–wave condition	Wind condition
Total length, L_OA_ (m)	148.00	4.606	2.467	4.606	2.467
Length between perpendiculars, L_pp_ (m)	140.30	4.3670	2.338	4.3670	2.338
Molded breadth, B (m)	23.40	0.7282	0.390	0.7282	0.390
Molded depth, D (m)	13.50	0.4201	0.225	0.4201	0.225
Draft, T (m)	8.00	0.2489	0.133	0.2489	0.133
Freeboard, *f* (m)	1.96	0.0610	-	0.0610	-
LCB (%LPP), fwd+	-1.48	-1.48	-	-1.48	-
Vertical Center of Gravity (from keel)	6.426	0.20	-	0.20	-
Moment of Inertia, K_xx_/B	0.40	0.40	-	0.40	-
Moment of Inertia, K_yy_/L_PP_, K_zz_/L_PP_	0.25	0.25	-	0.25	-
Area of orthogonal projection above waterline, A_T_ (m^2^)	519.6	-	0.144	-/0.502	0.144
Area of lateral projection above waterline, A_L_ (m^2^)	2173.3	-	0.603	-/2.105	0.603
Scaling ratio, *ε*	1	32.13	60	32.13	60

### 2.2 Wind tunnel tests for determining wind-load coefficients

**Fig 2:**
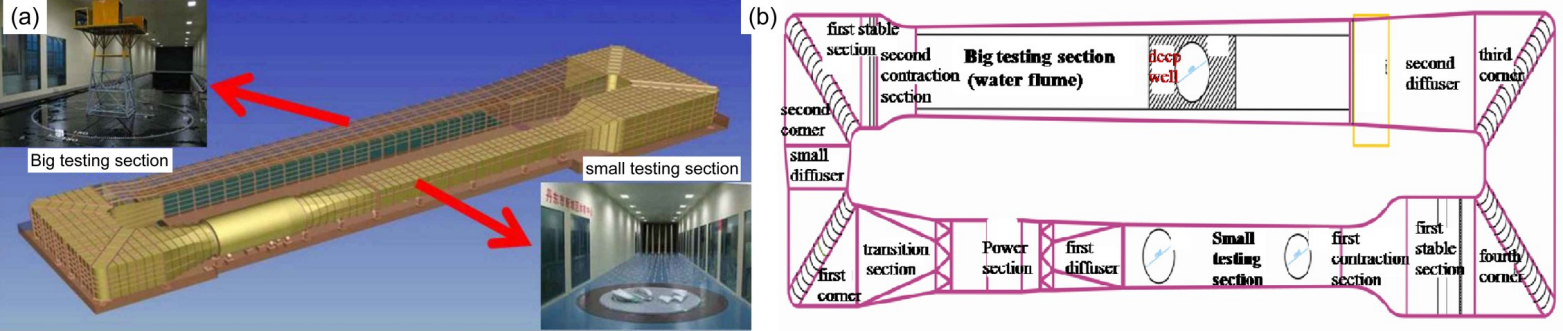
Geometry and schematic of the facility of wind tunnel combined with water flume at HIT. (a) Three-dimensional WTWF geometry; (b) WTWF schematic.

Wind-load coefficients for any ship can be approximated as constants with their values reflecting the shape and complexity of the ship superstructure. In this study, values of wind-load coefficients for the 1900TEU ship model were obtained via wind-tunnel experiments performed in the Wind Tunnel and Water Flume (WTWF) laboratory at the Harbin Institute of Technology (HIT) and installed with a closed single-return wind tunnel comprising two experimental sections—a large test section measuring 50 × 6 × 3.6 m (length × width × height) and a small section measuring 25 × 4 × 3 m—and a water flume—i.e., a tank measuring 50 × 5 × 4.5 m. Two automated rotating platforms, shown as circles in Fig 2(B), measuring 2.5 m in diameter, were installed within the smaller test section. A schematic of the wind-tunnel facility along with the water flume at HIT are shown in Fig 2. The wind-resistance test was performed inside the small test section. The experimental wind speed range is continuously adjustable from 3m/s to 50m/s (small test section) and 3m/s to 30m/s (large test section). The setup within the WTWF laboratory demonstrated excellent flow-field performance with non-uniformity, turbulence, and average airflow deviations within the experimental flow field measuring less than 1%, 0.46%, and 0.5°, respectively.

**Fig 3:**
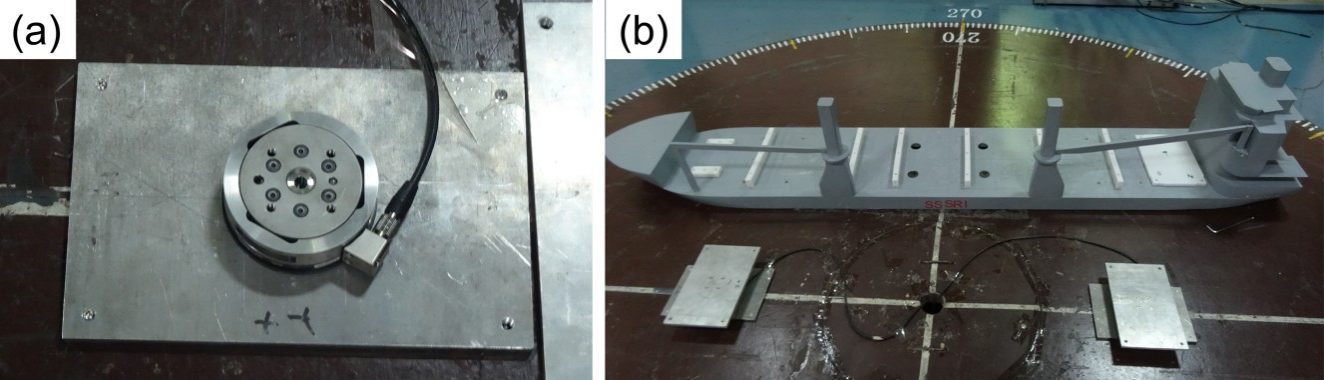
Dual-balance measuring mechanism—(a) High-accuracy six-component strain gauge balance; (b) experimental setup of dual-balance and test model.

Two high-accuracy, six-component strain-gauge balances were used during wind-tunnel experiments. With regard to the measurement range of the vertical-balance axis, the aforementioned strain-gauge balances could measure lateral forces (F_x_ and F_y_) of the order of 660 N; the corresponding measurable value of the axial force (F_z_) was 1980 N. Correspondingly, the range of the balance for lateral bending moments (M_x_ and M_y_) and torque (M_z_) was 60 Nm. The measurement error of the said balance was less than 1.0%. A dual-balance mechanism was used during measurements to prevent the load measured by a single balance from exceeding the above-mentioned maximum measurable values. The double balance and its connection with the ship model are shown in in Fig 3. The dual-balance setup was fixed at two ends around the center of a rotating platform with balances 1 and 2 being placed at distances *l*_*1*_ and *l*_*2*_, from the center, respectively. The ship model was placed horizontally inside the wind tunnel atop the dual-balance setup. The sum of the measured wind resistances obtained from both balances was recorded as the measured result during each trial. The corresponding wind-resistance-coefficient formulae are described using Eqs (1) and (2). A certain position was reserved for attachment of the balance to the model to reduce the distance between the bottom of the ship model and the rotating platform as much as possible. However, a certain clearance still existed, roughly equal to the thickness of the balance and measuring far less than the model height. The possible error source of the thickness has a negligible influence on the overall measurement result. The experimental setup of the wind tunnel considered in this study was identical to that described by Barcarolo D et. al. [18], who also considered the existence of the above-mentioned clearance between the ship-model bottom and rotating platform.

**Fig 4:**
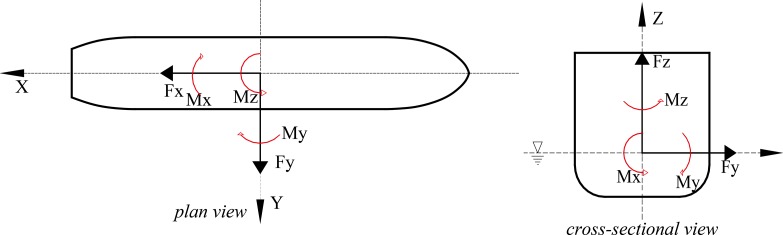
Axial coordinate system for describing wind forces acting on ship model.

**Fig 5:**
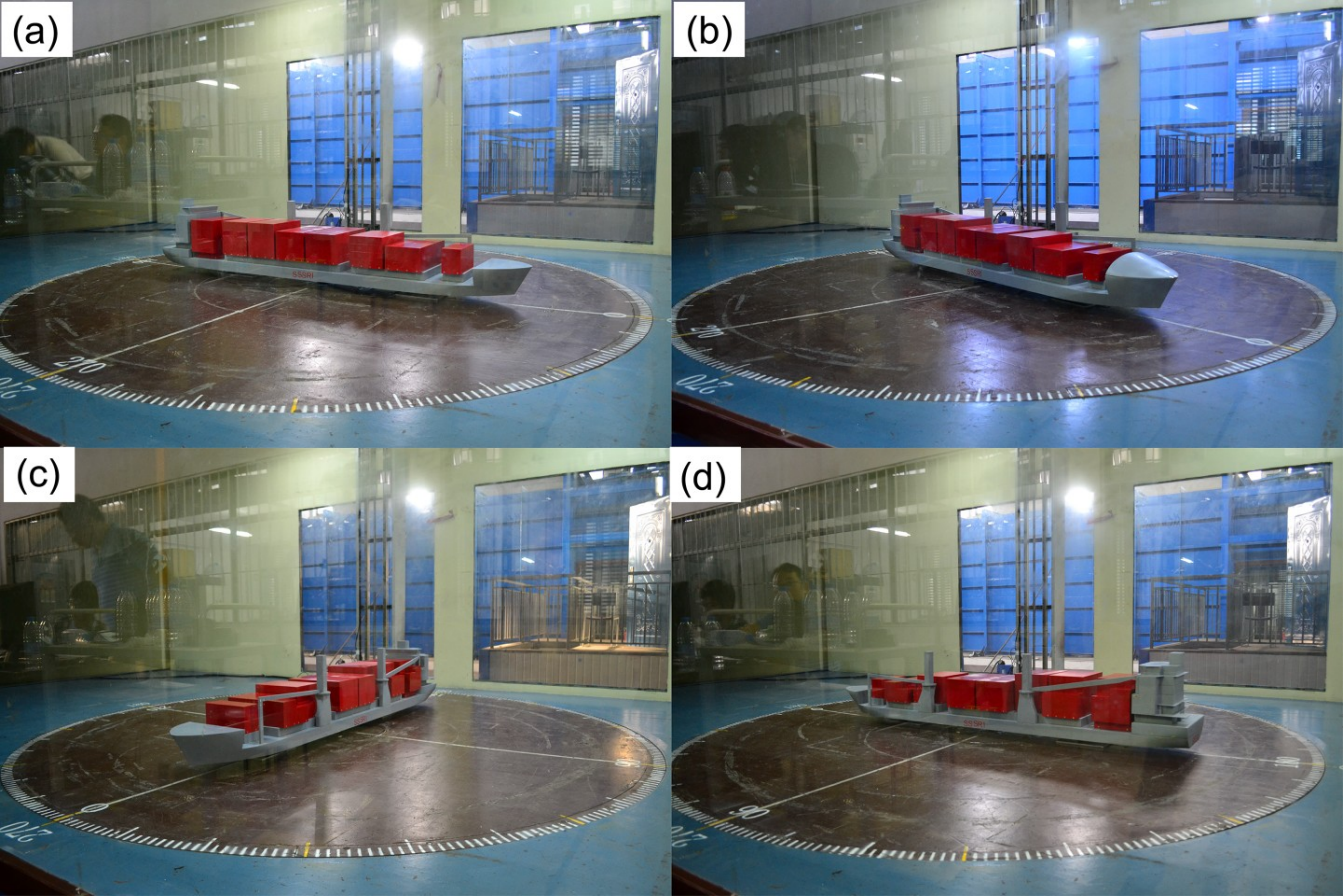
Different wind angles considered during the wind-tunnel experiment performed on the fully loaded container-ship model—(a) *θ* = 0°; (b) *θ* = 45°; (c) *θ* = 90°; (d) *θ* = 180°. Case *θ* = 0° implied wind direction to be aligned with ship bow.

Forces acting on the ship model during the experiment could be expressed in the coefficient form as follows:

$$C_{x}=\frac{F_{x}}{1/2\rho V^{2}A_{T}}=\frac{F_{x1}+F_{x2}}{1/2\rho V^{2}A_{T}}$$ (1)

and

$$C_{y}=\frac{F_{y}}{1/2\rho V^{2}A_{L}}=\frac{F_{y1}+F_{y2}}{1/2\rho V^{2}A_{L}},$$ (2)

where *F*_*x1*_, *F*_*x2*_, *F*_*y1*_, and *F*_*y2*_ denote the longitudinal- and transverse-force components measured using the two balances (subscripts "1" and "2" refer to balances 1 and 2, respectively); *A*_*T*_ and *A*_*L*_, respectively, denote areas of the frontal and lateral projections of the ship above the waterline. The inertial axial coordinate system for describing the actions of wind forces on the ship model is shown in Fig 4. The corresponding model parameters used for calculating wind-force coefficients are listed in Table 1. The critical Reynolds number [19, 20] of the flow was considered as the criterion to be satisfied during wind-tunnel experiments, and tests were performed under three wind speeds, 15 m/s, 20 m/s, and 25 m/s in accordance with the model scale. Once the critical Reynolds number criterion was satisfied by the wind speed, the corresponding wind-load coefficients under the three test conditions were observed to be nearly independent of the wind speed. Reynolds number can be expressed as Re = *ρUL*/*μ = UL/υ*, where *ρ* denotes fluid density, U denotes fluid velocity with respect to the model, L represents a characteristic linear dimension, and in this study L is the length of the water line of ship (Lwl) *μ* denotes dynamic viscosity of the fluid, and *ν* refers to the fluid kinematic viscosity. The average of three values of wind-load coefficients obtained for the three above-mentioned wind speeds was considered for further analysis. A set of photographs showing the experimental setup at different wind angles is shown in Fig 5.

### 2.3 Towing-tank experiment for measuring added resistance due to wave loads

**Fig 6:**
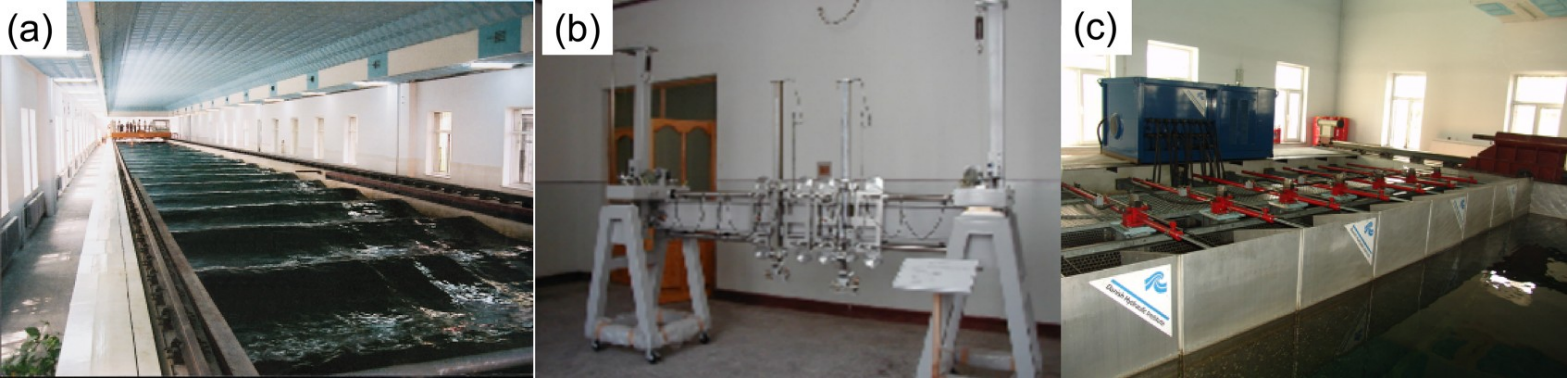
Setup for towing-tank experiments—(a) towing tank and towing-carriage system; (b) 4-component motion-measuring device; (c) 2D/3D wave maker.

The added resistance in wave condition was determined by performing towing-tank experiments. The ship model employed during these experiments corresponded to that of the 1900TEU container ship without a superstructure. Wave-resistance experiments were performed in the ship-model towing-tank laboratory at the Harbin Engineering University. The dimensions of the towing tank are 108 m in length, 7 m in width, and 3.5 m in depth. The towing carriage could operate at a maximum speed (V_max_) of 6.5 m/s, and was capable of handling speeds in the range of 0.1–6.5 m/s with a precision of 0.1%. Additionally, a 4-component motion-measuring device (GEL-421-1; Japan) equipped with heave and pitch sensors as well as a resistance dynamometer was used to measure both the resistance and motion parameters concerning the ship model. This device could measure resistance loads, heaves, surges, roll angles, and pitch angles of up to 150 N, ±200 mm, ±400 mm, ±50°, and ±50°, respectively, with a precision of 0.1%. The wave maker was capable of generating waves with amplitudes of the order of 0.4 m with periods varying from 0.4–4 s, with a precision of 0.1%. The experimental setup is shown in Fig 6.

**Fig 7:**
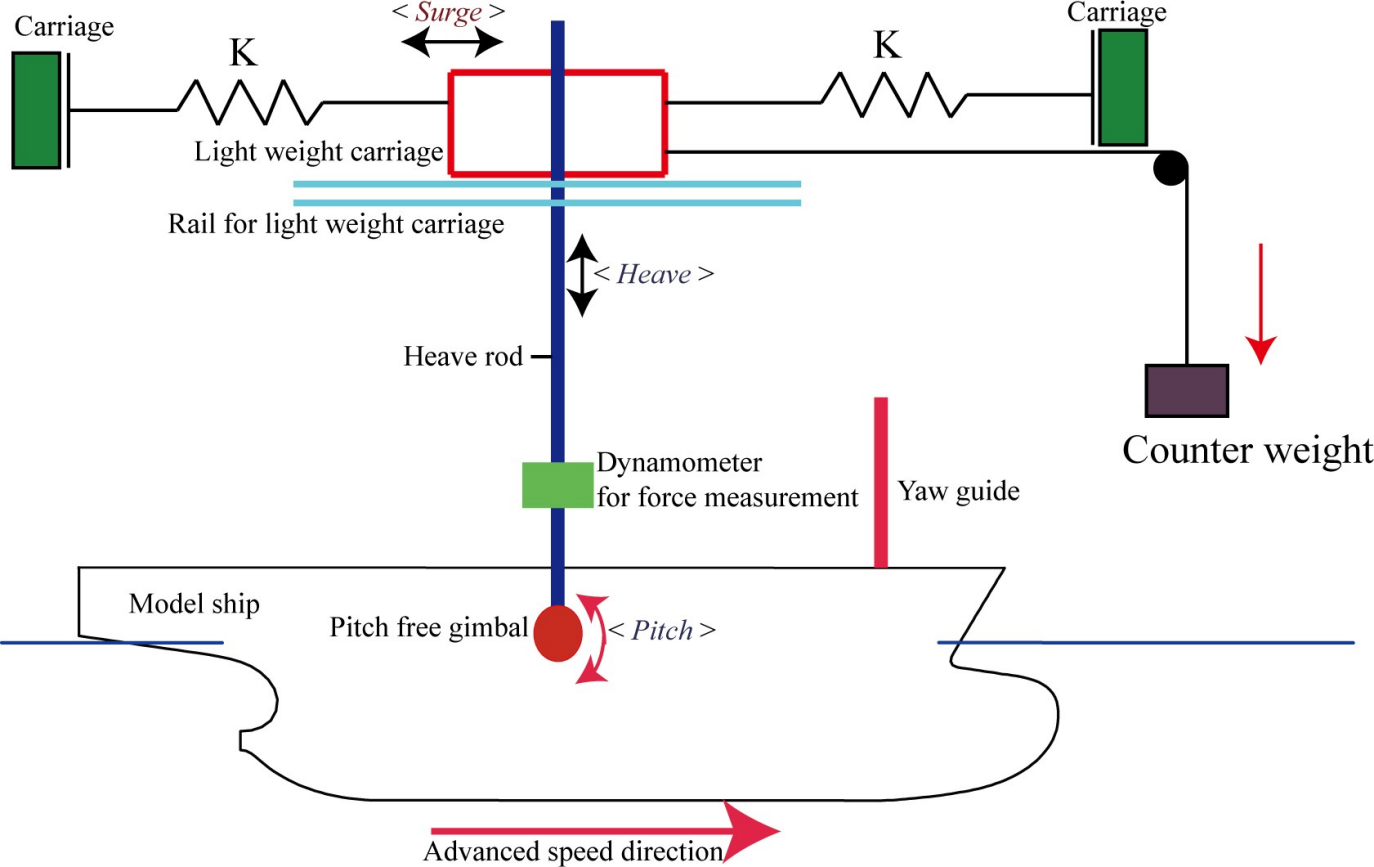
Schematic of experimental setup for measuring resistance and motion responses of ship model.

A schematic of the experimental setup for measuring the wave resistance and motion response of the ship model is shown in Fig 7. Force magnitudes were measured using a strain-gage-type load cell placed between the heave rod and pitch gimbal of the motion-measurement device. The 4-component device was used to tow the model and measure motion amplitudes whilst enabling free heave, pitch, surge, and roll motions. Degrees of motion of the ship motion could be adjusted by means of a simple lever-lock mechanism. Whilst performing measurements, the roll motion was locked, and only the heave and pitch motions were allowed. The measurement of heave motion is carried out at the center of gravity of the model. Towing-tank tests (under calm-water and wave conditions) were performed at five speeds corresponding to Froude number (F_r_) values in the range of 0.23–0.29 along with regular waves with wave height (*H*_*w*_) measuring approximately 0.08 m and wavelength (λ) of the order of 0.25 *L* and 0.5 *L*. The Froude number can be expressed as $Fr=U/\sqrt{gL}$, where *U* denotes fluid velocity with respect to the model, *g* denotes acceleration due to gravity, and *L* refers to the model length at the waterline level. The main purpose behind selection of wave parameters during experimental and numerical calculations was to investigate trends in the isolated wave resistance and resistance offered under combined wind and waves load whilst considering the same wave height and different wavelengths. The method of selection of wave parameters was the same as that in available literature [21–24].

## 3. Numerical modeling and CFD mesh setup

### 3.1 Governing equations

The unsteady Reynolds-averaged Navier–Stokes (URANS) approach yields reasonably accurate numerical results and performs reliable flow-field simulations for facilitating hydrodynamic and aerodynamic analyses of ships (with and without superstructures) with reasonable mesh sizes and within acceptable computation time [25–27]. This approach was, therefore, considered for use in numerical simulations performed in this study. The motion-compliant continuity and momentum-conservation equations [28] can be written as

$$\frac{\partial \rho }{\partial t}+\frac{\partial \left(\rho u_{i}\right)}{\partial x_{i}}=0$$ (3)

$$\frac{\partial \left(\rho u_{i}\right)}{\partial t}+\frac{\partial }{\partial x_{j}}\left(\rho u_{i}u_{j}\right)=-\frac{\partial p}{\partial x_{i}}+\frac{\partial }{\partial x_{j}}\left(\mu \frac{\partial u_{i}}{\partial x_{j}}-\rho \bar{u_{i}^{\prime }u_{j}^{\prime }}\right)+S_{j}$$ (4)

where *u*_*i*_ and *u*_*j*_ (*i*, *j* = 1, 2, 3) denote time-averaged values of velocity components; *p* denotes time-averaged pressure; *ρ* denotes fluid density; $\rho \bar{u_{i}^{\prime }u_{j}^{\prime }}$ refers to the Reynolds stress term; and *S*_*j*_ denotes the source term. Wind speeds of the order of 15, 20, and 25 m/s (Mach number Ma = 0.042, 0.059, and 0.0745), which are less than 110 m/s (Ma = 0.33), as reported by Som et. al. [29], were considered. The fluid density was assumed constant for incompressible Newtonian fluids.

### 3.2 Turbulence model and free-surface treatment

The finite-volume computational method in combination with a segregated flow solver was used to analyze the 1900TEU container-ship hull. The solver used was pressure-based and fully coupled with second-order upwind spatial and central discretization for convective-flux and diffusion terms, respectively. An implicit pseudo-time-marching scheme was used to attain convergence. Preconditioning ensures suitability of use of the URANS approach for simulating low-speed, isothermal flows [30]. A point-implicit (Gauss–Seidel) linear system solver with algebraic multigrid acceleration was used to solve the resulting discrete linear system of equations during each iteration. The shear stress transport (SST) *k*-*ω* turbulence model [31] was employed for numerical simulations, because it can effectively deal with the strong freestream sensitivity of the *k*-*ω* turbulence model, thereby improving the prediction accuracy of adverse pressure gradients. Free-surface of the ocean was modeled using the two-phase volume-of-fluid (VOF) technique [32].

### 3.3 Numerical wave generation and wave damping

The STAR-CCM+ commercial software package was used to perform numerical wave simulations. Wave generation in the STAR-CCM+ solver was accomplished using the inlet and outlet boundaries, at which incident-wave boundary conditions were enforced. Specifically, this approach (referred to as the boundary velocity input method) sets a velocity profile over the water depth of waves at the inlet boundary. Variables concerning wave motion could be expressed as [33]

$$Horizontal\;velocity:\;v_{h}=a\omega \cos(K\cdot X-\omega t)e^{Kz}$$ (5)

$$Vertical\;velocity:\;v_{v}=a\omega \sin(K\cdot X-\omega t)e^{Kz}$$ (6)

Surfacewaveheight:η=acos(K⋅X−ωt) (7)

where *a* denotes wave amplitude; ω represents angular frequency in radians/s; **K** refers to the wavenumber vector; *K* denotes magnitude of the wavenumber vector; and z represents vertical distance from the mean water level.

$$\mathrm{Wave}\;\mathrm{period}:\;T=\frac{2\pi }{\omega }$$ (8)

$$\mathrm{Wavelength}:\;\lambda =\frac{2\pi }{K}$$ (9)

Numerical head-wave simulations were performed considering a wave height *H*_*w*_ = 0.08 m and wavelengths *λ* = 0.25 *L* and *λ* = 0.5 *L*.

To avoid wave reflection, a numerical wave beach with *l* = 2λ damping area was established at the outlet boundary of the tank to facilitate wave damping by providing resistance against vertical wave motion. The method devised by Choi and Yoon [34] prescribes the addition of a resistance term to the equation for *w* as follows.

$$S_{z}^{d}=\rho \left(f_{1}+f_{2}\left|w\right|\right)\frac{e^{\kappa }-1}{e^{1}-1}w$$ (10)

with

$$\kappa ={\left(\frac{x-x_{sd}}{x_{ed}-x_{sd}}\right)}^{n_{d}}.$$ (11)

In the above equations, *x*_*sd*_ denotes the starting point for wave damping (for waves propagating along the *x*-direction), and *x*_*ed*_ denotes the corresponding end point (boundary); *f*_1_, *f*_2_, and *n*_*d*_ refer to parameters concerning the damping model, the values of which were set to 10, 10, and 2, respectively, in this study; lastly, *w* denotes the vertical velocity component.

### 3.4 Dynamic fluid–body interaction model for rotational and translational motion

The dynamic fluid–body interaction (DFBI) module within STAR-CCM+ simulates motion of a rigid body in response to pressure and shear forces exerted by fluid motion along with any additional forces defined by the user. The solver calculates values of resultant forces and moments acting on the body owing to all influences considered. It also solves governing equations for rigid-body motion to determine the new position of a rigid body. Thus, dynamic ship motions, including surge, sway, heave, roll, pitch, and yaw, can be numerically analyzed using the DFBI module employing a six-degrees-of-freedom (DOF) system.

Motions of the ship model considered in this study were described in terms of earth- and ship-fixed inertial reference coordinate frames. The flow field was solved in the earth-fixed coordinate system because operating conditions considered in this study comprised head-sea waves aligned with the freestream wind direction sans parametric roll motion. Therefore, only heave and pitch motions were considered in this study. Ship motion was described in terms of translation and rotation with respect to the earth-fixed frame as well as linear and angular velocities and corresponding forces and moments acting in the ship-fixed frame. Details concerning the 6-DOF module have previously been described by Carrica et al. [35–36].

### 3.5 Simulation of wind gradient and fluctuations

Generally, the study of ship wind load is only loaded with the static load of time-averaged wind, but the distribution of unsteady wind in space and time is more complex in actual sea conditions. There is a simplified error in the performance evaluation of real ship under sea conditions when the results of the static load method are used to study the wind load.As the ocean wind field is not obstructed by obstacles, and because roughness of the sea surface is relatively low, the wind speed gradient tends to increase along the vertical direction normal to the sea surface. Based on terrestrial and marine wind-field distributions recorded in Europe, it has been inferred that within the same region, average wind speeds near seas measure at least 25% higher compared to terrestrial wind speeds. The wind profile of a wind field is approximately related to its height above sea level or land surface in accordance with the following equation.

$$V_{z}=V_{z_{0}}{\left(\frac{z}{z_{0}}\right)}^{\alpha }$$ (12)

Here, *V*_*z*0_ denotes the reference wind speed at a height *z*_0_ above the sea surface; *V*_*z*_ represents the corresponding wind speed at height *z*; and *α* denotes the wind shear index, its value lying in the range of 0–1 and set to 0.1 in the proposed study. The gradient value reflects changes in wind speed near the sea surface. Wind speeds generally approach zero around the free surface of seas owing to the action of fluid viscosity. Between the sea surface and reference height, the wind speed increases with a large gradient. Above the reference height, the aforementioned gradient assumes a small value.

Wind loads under actual oceanic conditions can be decomposed into average winds and wind fluctuations. Fluctuating winds are three-dimensional, unsteady, and turbulent, and include tailwinds (blowing in the direction of ship motion), cross winds (blowing horizontally and perpendicular to the ship centerline), and vertical winds (blowing along the vertical direction perpendicular to the ship centerline).

In this study, wind gradients and wind fluctuations were used to simulate actual oceanic conditions of wind fields at sea. A height of 10 m above the sea level (i.e., model scale height of 0.19 m) was considered as the reference height for calculating wind gradients with values of *V*_*Z*0_ and *V*_10_ being set as 15 m/s to satisfy the critical Reynolds number criterion and facilitate comparison against results obtained via wind-tunnel experiments. The simulation of wind fluctuations was simplified by dividing these velocities into averaged and fluctuating components are expressed as

$$\begin{array}{l} V(x,y,z,t)=\bar{V}(z)+v(x,y,z,t)\\ v(x,y,z,t)=\left(v_{x}(x,y,z,t),v_{y}(x,y,z,t),v_{z}(x,y,z,t)\right) \end{array}$$ (13)

In this study, components of only the fluctuating wind field along the tailwind direction were considered, and stochastic processes of wind fluctuations under actual oceanic conditions were neglected. Thus, actual 3D wind fields were simplified to 1D wind fields, and the graded distribution (with *α* = 0.1) and fluctuating components along the vertical direction were simplified as a cosine distribution. The fluctuation period was defined as the period of ship motion *T*. The selection of the fluctuation period in this paper is a kind of numerical simplification. And since the research focus of this paper is the influence of wave and wind-wave coupling environment on force and motion of the ship, in order to ensure that the action of wind gust on the ship corresponds to the motion of the ship, the fluctuation period was defined as the period of the ship motions in the waves. Through the experiment and numerical simulation results of ship motion in waves, we can obtain the period of ship motion in waves. The wind gust input is carried out through the boundary conditions of velocity inlet with the corresponding wind gust equation., and the amplitude of a fluctuating wind at the reference height was set to 5 m/s.

$$V(z,t)=V_{z_{0}}{\left(\frac{z}{z_{0}}\right)}^{0.1}+v_{x}\left(z,t\right)=V_{z_{0}}{\left(\frac{z}{z_{0}}\right)}^{0.1}+2.5\mathrm{lg}(\frac{z+z_{0}}{z_{0}})\cos\left(\frac{2\pi }{T}t\right)$$ (14)

Therefore, the fluctuating wind function *V*(*z*, *t*) was used to numerically simulate a fluctuating wind field, and the fluctuating winds were generated at the inlet boundary of the computational zone.

### 3.6 Overset mesh for CFD calculations

**Fig 8:**
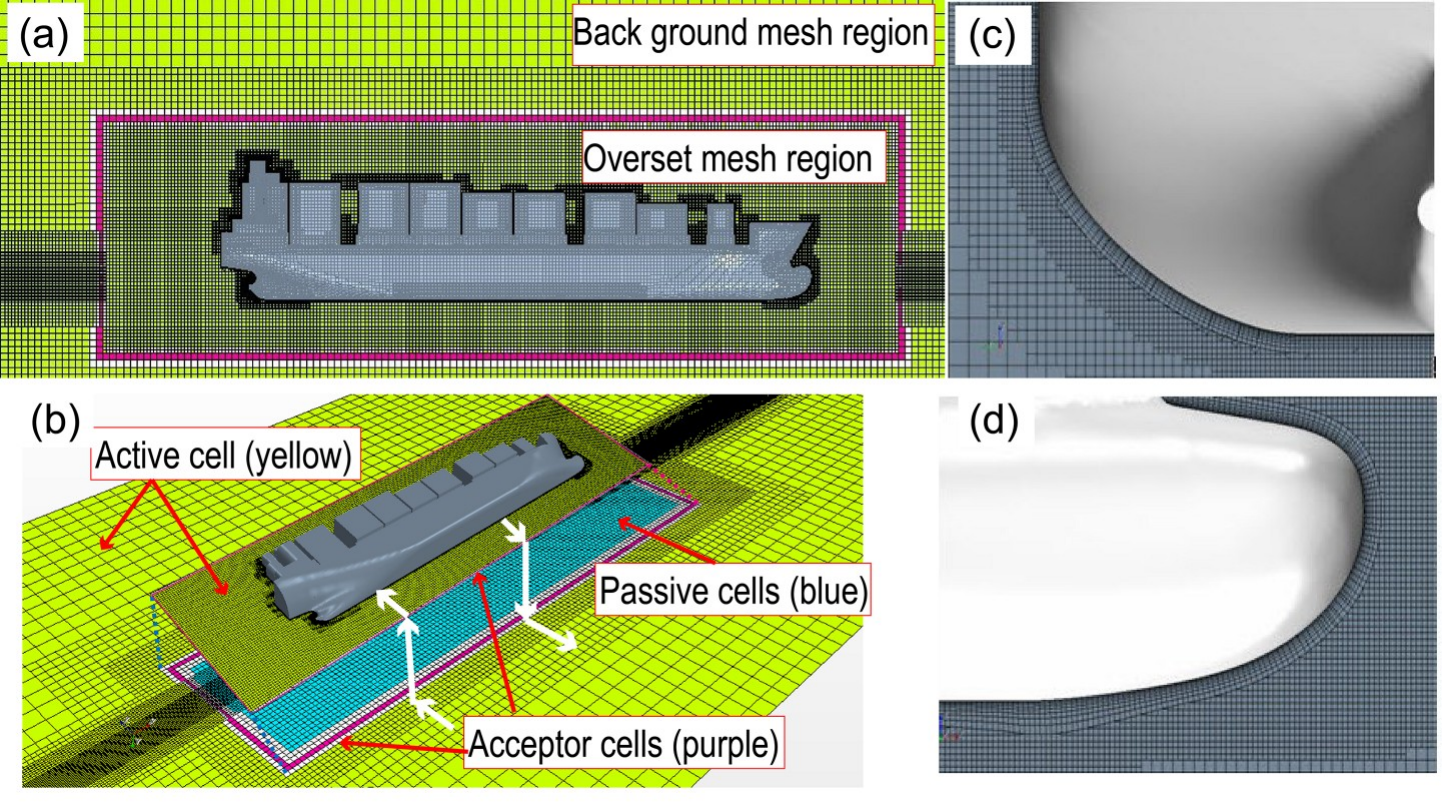
CFD mesh—(a) entire mesh; (b) overset mesh; (c) stern mesh; (d) bow mesh.

Under the action of waves that exhibit large deformations at the free surfaces, ships usually perform motions with large-amplitudes. With increase in ship amplitude, the use of traditional dynamic meshing methods results in abnormal deformation and low efficiency of grid reconstruction. These limitations of the dynamic meshing technique in handling large motions can be overcome using overset meshes that provide an effective solution to nonstationary problems. Illustrations of an overset mesh are shown in Fig 8. The trimmed mesh includes a boundary layer along with an overset mesh with the boundary layer being set to be consistent under calm-water conditions. To facilitate the capturing of waves in greater detail, a minimum of 80 mesh elements were used within the range of each wavelength, and the wave amplitude of each wave contained at least 20 mesh elements.

### 3.7 Numerical convergence assessment

The assessment of numerical convergence is mainly conducted in two parts. The first part is the numerical setting part. The most important factor affecting the selection principle of time step and the basis of convergence is the Courant–Friedrichs–Levy number $CFL=\frac{U\Delta t}{\Delta x}$, CFL is Courant–Friedrichs–Levy number, *U* is reference speed, for ship *U* is ship speed, for wave *U* is group speed *C*_*g*_, $C_{g}=\frac{1}{2}\sqrt{\frac{g\lambda }{2\pi }}$. *g* is gravitational acceleration, *λ* is wavelength, Δ*t* is time step, Δ*x* is grid size. In order to capture all perturbations in physics, the velocity of physical perturbation propagation of the difference equation is less than that of time-marching solution. For the CFD calculation process with a free surface, it was important that the time-step size satisfied the demands of the Courant–Friedrichs–Levy number CFL < 1. Ten inner iterations were used for the convergence of the flow field equations within each time step. The second part is the convergence monitoring of numerical calculation, which mainly includes the monitoring of residual, physical quantity and flow field data. The following criteria are used to determine whether a simulation has converged: 1) In a converged solution, it would be expected that residuals would drop at least a couple of orders of magnitude (a good rule of thumb for them to drop 3 orders of magnitude); 2) In order to judge convergence, physical quantities must also be tracked, and, through reporting and plotting such quantities, convergence can be assumed when the engineering quantities have reached an unchanging state or have stable periodic changes under numerical conditions with periodic changes; 3) Creating scalar and/or vector plots of the entire solution domain or parts of it, and visualizing these plots as the solution develops, is also helpful in judging convergence; by observing any changes in the flow field. This technique can also be useful when comparing to residuals that cannot be exhibiting a converged solution. In this case, visualizing the solution and observing any changes in the flow field that are happening at a location in the domain far from any geometry or solution feature of interest, can help determine that a satisfactory solution has been obtained. In the wind load numerical simulation and added resistance due to combined wind-wave loads numerical simulation part of the paper, through the results of residual, monitoring engineering quantity and visualizing the flow field, it is shown that the numerical part of this paper is convergent.

## 4. Computational results and analysis

### 4.1 Study of wind loads

**Fig 9:**
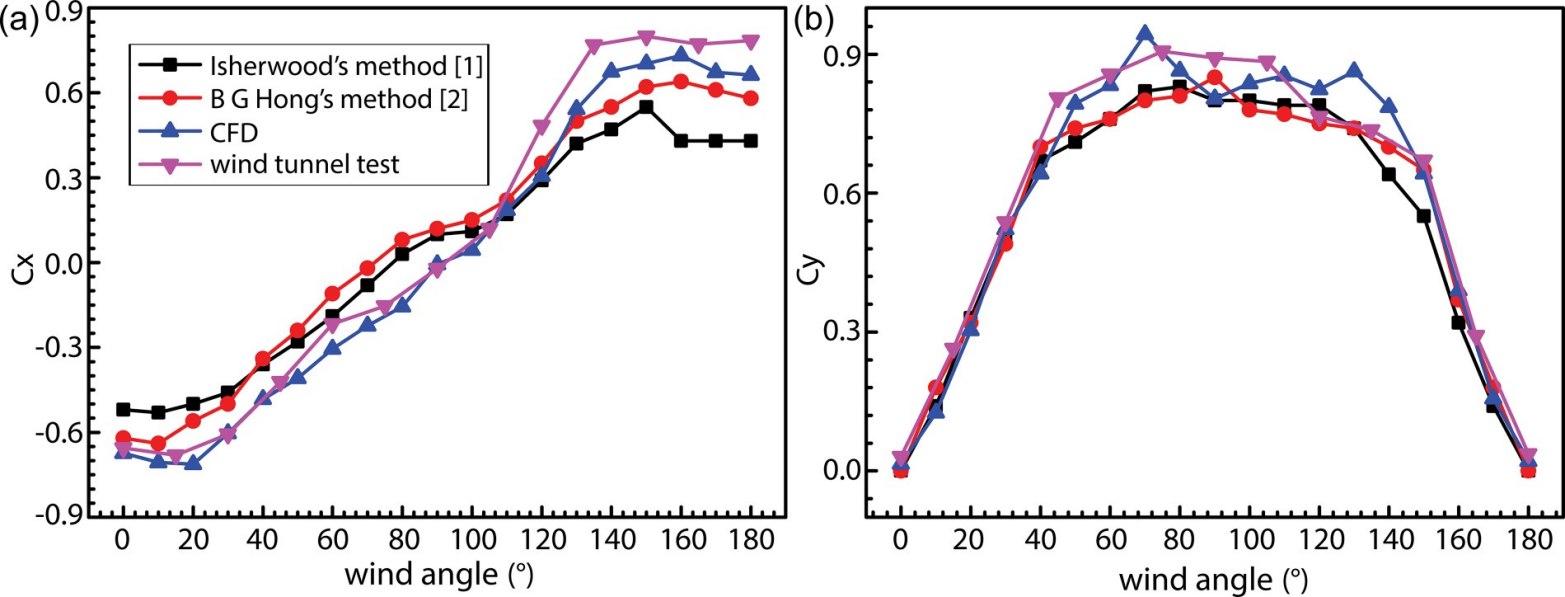
Comparison between experimental and calculated values of longitudinal (*C*_*X*_) and transverse (*C*_*Y*_) wind-load coefficients.

A comparison of wind-load coefficients obtained using empirical calculations prescribed in models proposed independently by Isherwood and Hong, CFD calculations, and wind-tunnel experiments under conditions of different wind angles are shown in Fig 9. In accordance with Isherwood’s method [1], wind-tunnel test data for 11 ships were considered as samples, and six ship-type parameters were extracted from experimental wind-tunnel data to be used as independent variables for multiple linear regression. Regression coefficients were, thus, obtained for wind angles in the range of 0–180° at 10° intervals. These regression coefficients were subsequently used to acquire longitudinal and lateral wind-load coefficients and the yawing moment at different wind angles. Based on this, an empirical equation for calculation of wind-load coefficients was proposed via wind-tunnel experiments and regression of experimental data obtained for actual ships. Variables considered in this approach are mutually independent and include basic parameters concerning each ship type with most major ship types being sampled, thereby providing a high degree of computational accuracy. In accordance with the method prescribed by B. G. Hong [2], Fourier-series expansions were performed on wind-load coefficients acquired from wind-tunnel data concerning 50 ship models, thereby obtaining fifth-order harmonic expressions as functions of the wind angle. In particular, Fourier coefficients were obtained by solving a coefficient matrix obtained via multivariate quadratic regression. This method offers a high degree of computational accuracy when compared with Isherwood’s method [1] and experimental results. Hong’s method can, therefore, be used for rapid prediction of wind-load coefficients with an extremely high level of accuracy in the prediction of longitudinal load coefficients at small wind angles. As observed in Fig 9, values of longitudinal wind-load coefficients obtained via CFD are only slightly different from those determined experimentally; i.e., the CFD curve effectively coincides with the experimental curve at smaller wind angles with a slight divergence at wind angles exceeding 150°. Values of wind-load coefficients obtained from wind-tunnel experiments are generally slightly higher compared to those obtained via CFD. Values of transverse wind-load coefficients (for wind angles in the range of 0–40° and 140–180°) calculated using empirical correlations and CFD demonstrate only slight differences. However, these values demonstrate varying degrees of divergence from those obtained experimentally at wind angles in the range of 40–140°, wherein values obtained using Hong’s method are most similar to those obtained via wind-tunnel experiments. Wind-tunnel experiments are amongst the most reliable methods for studying wind-loads acting on ship structures. The results of these experiments and those obtained via CFD calculations and empirical methods are different for each procedure. Overall, values obtained from CFD calculations were observed to demonstrate the highest agreement with those determined experimentally. Nonetheless, Hong’s method—useful for rapid wind-load predictions—demonstrated an extremely high level of accuracy, with regards to the prediction of longitudinal wind-load coefficients at small angles. Compared to experimental results, errors incurred when employing Hong’s method were observed to have maximum values of 10.8% and 8%, respectively, when calculating longitudinal wind-load coefficients within the wind-angle range of 0–40° and transverse wind-load coefficients at wind angles of 0–180°.

**Fig 10:**
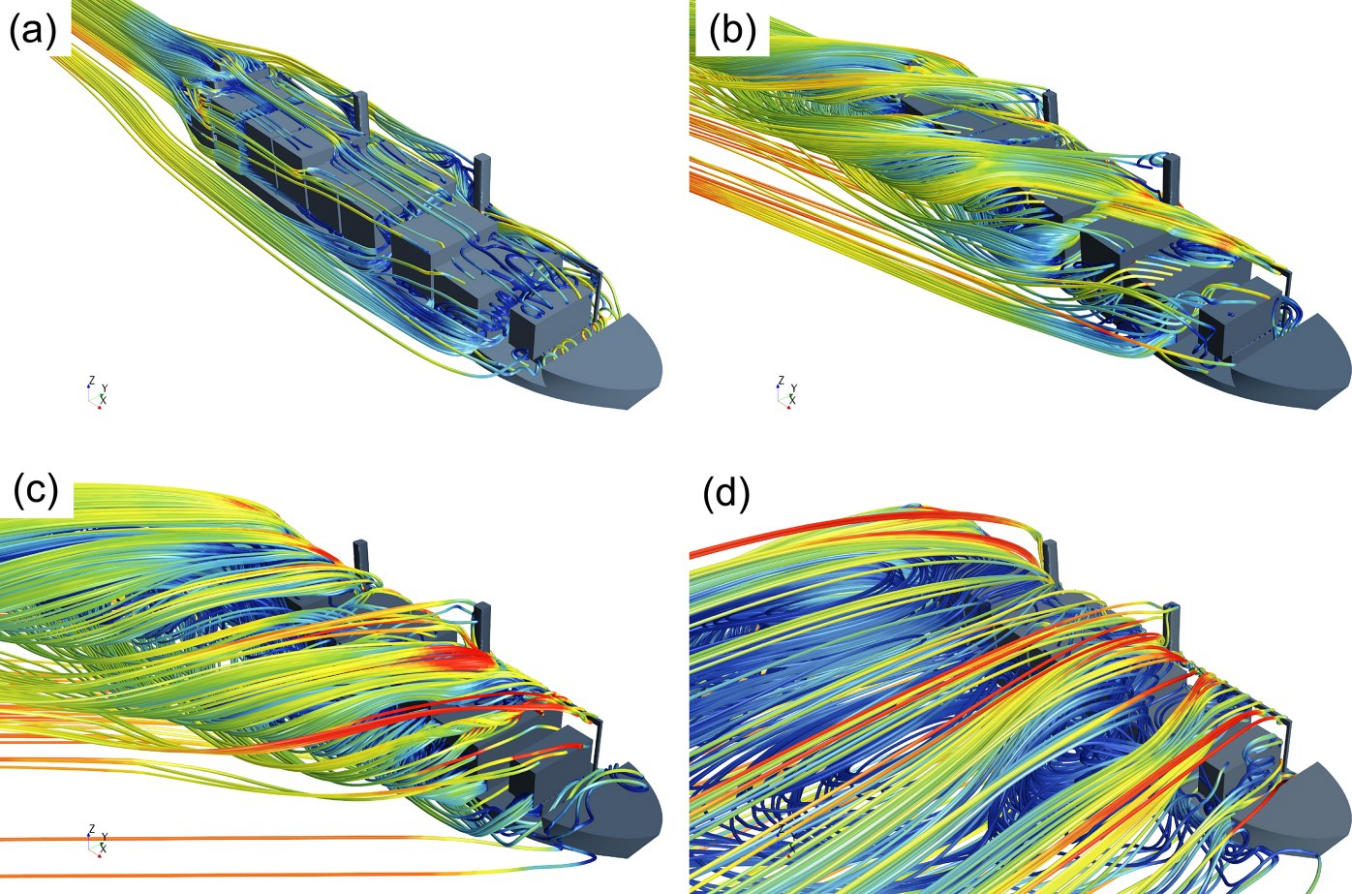
Numerical streamlines for 1900TEU container ship set at different wind angles—(a) *θ* = 0°; (b) *θ* = 20°; (c) *θ* = 50°; (d) *θ* = 90°.

**Fig 11:**
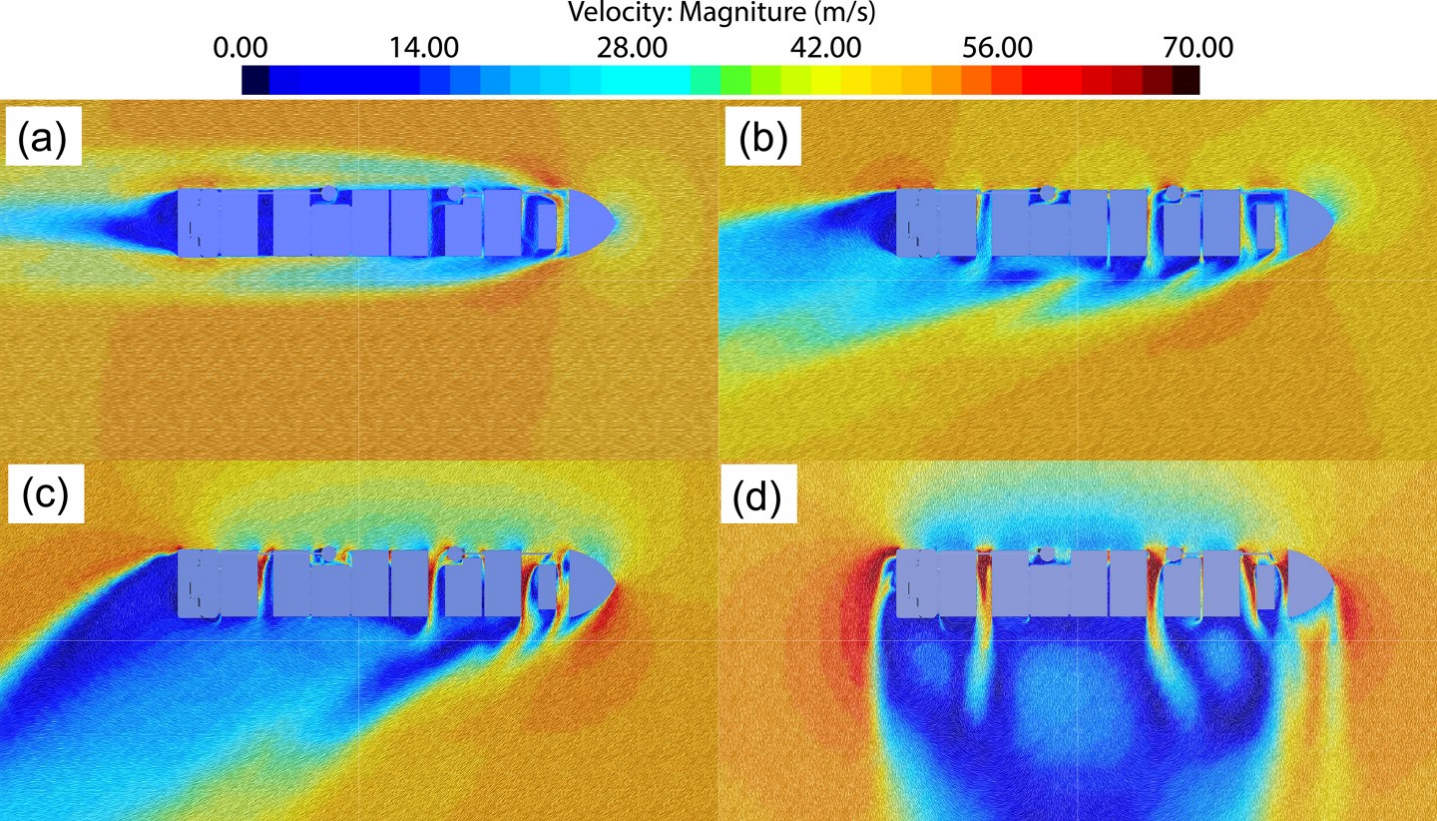
Distribution of numerical vortex traces on a 1900TEU container ship set at different wind angles—(a) *θ* = 0°; (b) *θ* = 20°; (c) *θ* = 50°; (d) *θ* = 90°.

**Fig 12:**
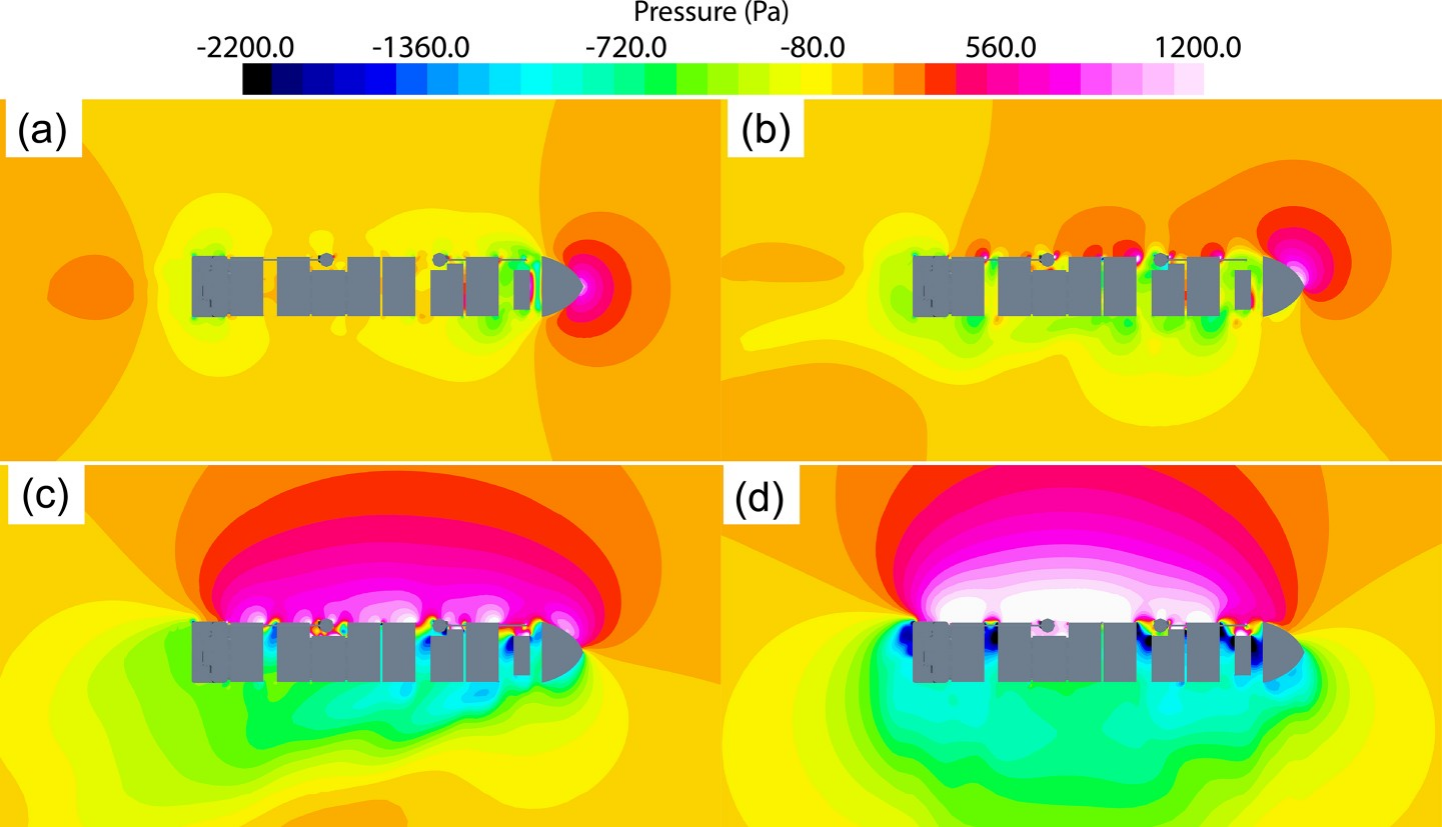
Distribution of numerical wind pressure around a 1900TEU container ship set at different wind angles—(a) *θ* = 0°; (b) *θ* = 20°; (c) *θ* = 50°; (d) *θ* =90°.

Figs 10–12 show numerically obtained distribution of streamlines, vortex traces, and pressure fields around superstructure containers at different wind angles. Fig 10 shows that when the wind angle *θ* = 0°, detached eddies are generated when the incoming flow detaches from the bow and superstructure. Additionally, compared to the bow region, there exists a low-pressure zone at the stern. As the wind angle increases in the range of *θ* = 20–50°, detached eddies tend to form along the leeward side, and the severity of flow separations on this side results in creation of a large detached-eddy zone. At *θ* = 90°, transverse winds get obstructed by containers and the superstructure. The air viscosity causes incoming flows to disperse along the ship superstructure, thereby resulting in creation of very large detached-eddy zones (the largest among the cases considered) on the leeward side.

From Figs 11 and 12, we observe that as the wind angle is increased, the ship model tends to get increasingly affected by lateral winds, thereby resulting in stronger flows around the superstructure and expansions within the low-pressure, detached-eddy zone on the leeward side. With increase in wind angle from *θ* = 0° to *θ* = 90°, both the magnitude and effective area of wind-pressure action on the windward side tend to increase, whereas values of corresponding parameters on the leeward side reduce, thereby resulting in growing differences in wind pressure between the windward and leeward sides. Therefore, it can be inferred that values of transverse wind-load coefficients for ship structures generally exceed those of longitudinal ones.

### 4.2 Investigation of added resistance due to wave loads

**Fig 13:**
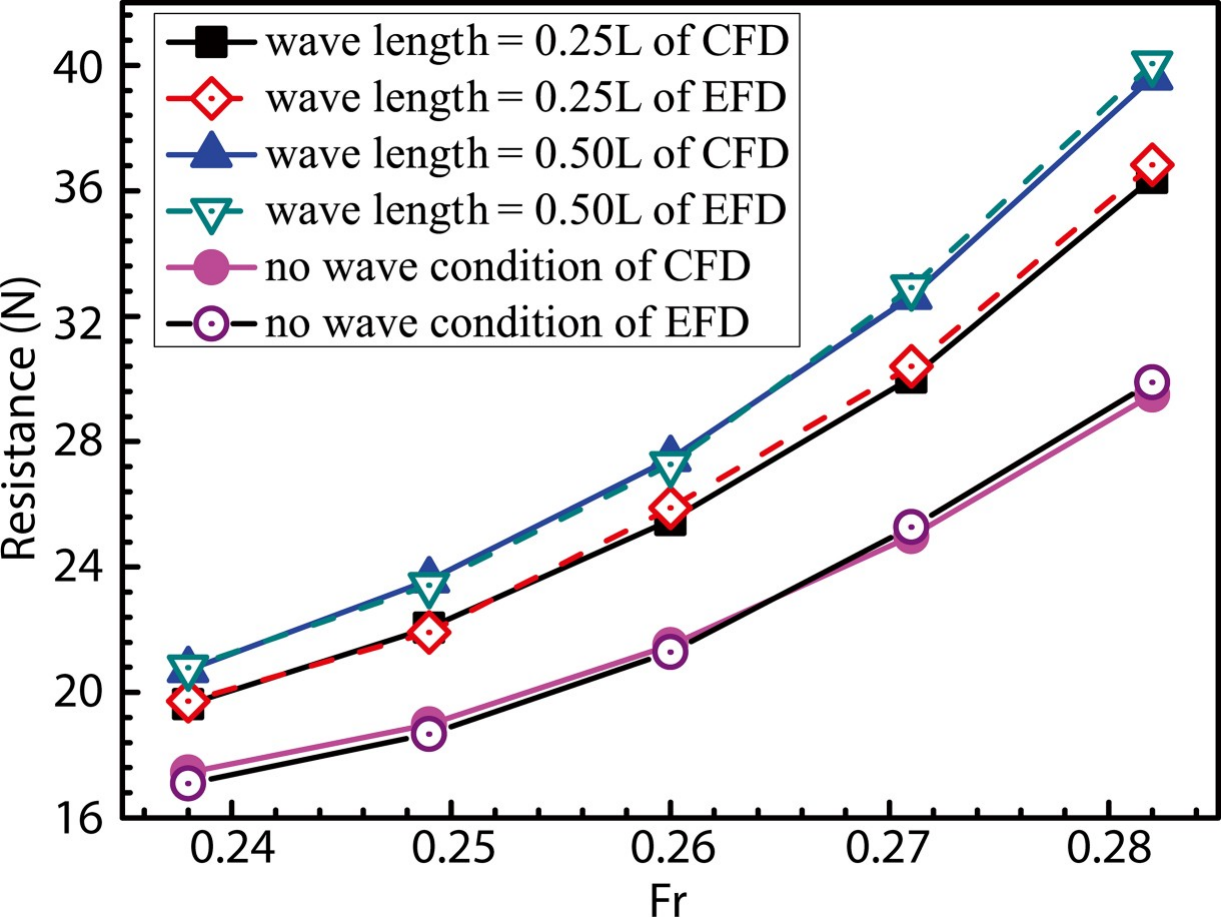
Relationship between resistance and ship speed under calm-water and wave conditions.

Relationships between resistance and ship speed under calm-water and a variety of wave conditions are shown in Fig 13. Under calm-water conditions, the CFD-calculated value of resistance nearly equals that obtained experimentally. Under wave conditions, CFD results generally demonstrate good agreement with experimental trends, especially at low ship speeds, with error values measuring less than 1.5%. With increase in ship speed, CFD-calculated values tend to become smaller compared to those determined experimentally. Under the condition for which F_r_ = 0.28, maximum errors of the order of 4.05% and 4.12% were observed to occur at wavelengths of 0.25*L* and 0.50*L*, respectively. In addition, the ship resistance was observed to be significantly higher under wave conditions compared to the calm-water case, and the added resistance due to wave loads increased with increase in ship speed. For the added-resistance in waves, this should be mostly a second-order potential-flow effect for the studied wavelength-to-ship length ratios, in the range of short wavelengths (*λ*/*L<*1), the wave added- resistance was observed to increase with increase in the value of *λ*/*L* and attain maxima at *λ*/*L* = 1 [37–38].

**Fig 14:**
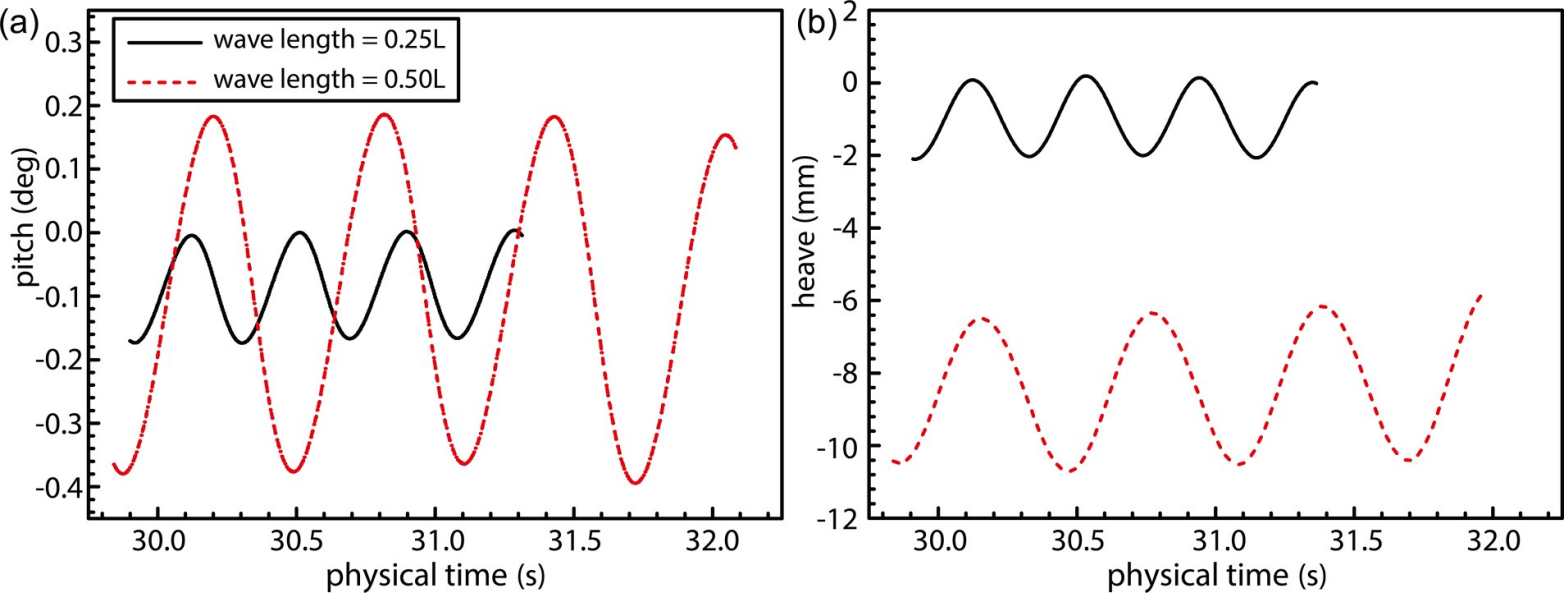
Time-history curves for numerical pitch and heave under wave conditions with Fr = 0.26.

Fig 14 shows the temporal changes in the ship-motion characteristics under wave conditions corresponding to Fr = 0.26 and wavelength ratios of 0.25 and 0.50. The added resistance of ship motion caused by wave is an important component of the total ship resistance in wave Fig 14 shows periodic patterns exhibited by the pitch and heave motions. The pitch curve (Fig 14(A)) indicates the occurrence of head trim under wavy conditions, thereby resulting in the bow dipping under water. The average head trim of the ship under wavelengths of 0.50*L* and 0.25*L* was observed to be similar. Heave curves, on the other hand, indicate existence of a greater draft at 0.50*L* compared to that at 0.25*L*, and this constitutes a large component of the added resistance. The observed heave was greater in magnitude at 0.50*L*, thereby exacerbating the ship motion. The mean values heave of λ/L = 0.25 and λ/L = 0.50 wave conditions in Fig 14(B) are composed of two parts. The first part is the sinkage values caused by the hydrodynamic pressure corresponding to the ship speed, literature [39]; the second part is the ship heave caused by wave surface under the wave conditions. When the ship sails in calm water with a higher speed, the container ship will in the state of sinkage. The sinkage value is related to the hydrodynamic pressure distribution around the ship. When the ship sails in wave condition, the wave surface of λ/L = 0.25 condition has a more significant degree of tortuosity than λ/L = 0.50 wave condition, and the hydrodynamic pressure distribution around the hull under λ/L = 0.50 wave condition is more stable than λ/L = 0.25 condition. So the sinkage value caused by the hydrodynamic pressure under λ/L = 0.25 condition is small than λ/L = 0.50 wave condition. In addition, the wave steepness under the condition of λ/L = 0.25 is more remarkable than that under the condition of λ/L = 0.50, and the non-linearity of the wave is more remarkable, and the free surface of the corresponding linear wave is raised. At this time, the average heave value is larger under the condition of λ/L = 0.50.

### 4.3 Investigation of added resistance due to combined wind-wave loads

**Fig 15:**
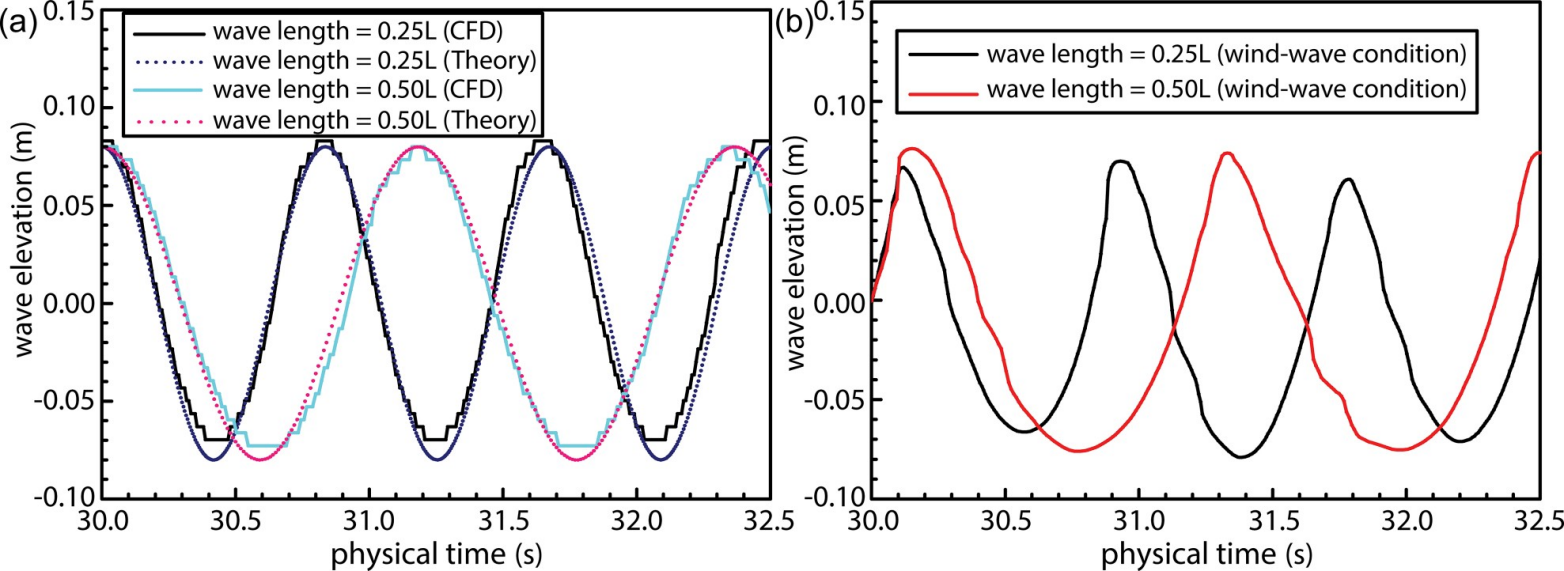
Results obtained via numerical simulation of wind and wave loads—(a) Time history of wave surface under isolated wave loads; (b) Time history of wave surface under combined wind–wave loads.

During simulation of combined wind–wave loads, the direction of waves and the wind were considered to be aligned with the ship bow. Additionally, interactions and the coupling between wind- and wave-induced loads were considered to affect wave parameters, thereby altering the additional resistance faced by the ship. Prior to calculating the added resistance under combined presence of wind and wave loads, loads acting on the ship were simulated under conditions of combined wind–wave action and wave loads alone. This allowed the authors to ascertain the accuracy of wind- and wave-load simulations. As shown in Fig 15(A), changes in the wave surface during wave load alone demonstrate a good fit with the theoretical curve with peak values exactly coinciding theoretically determined ones, whereas trough values were observed to be slightly lower compared to those predicted theoretically. Overall, the simulated wave field demonstrated an adequate level of accuracy. Fig 15(B) illustrates results obtained for simulated wind and wave loads. In this figure, wave parameters can be observed to have been altered by the action of turbulent wind fields on the wave surface. Consequently, peak values were observed to increase relative to those shown in Fig 15(A), and lengths of the crest and trough regions can be observed to have been shortened and lengthened, respectively, thereby resulting in sharper peaks and rounded troughs.

**Fig 16:**
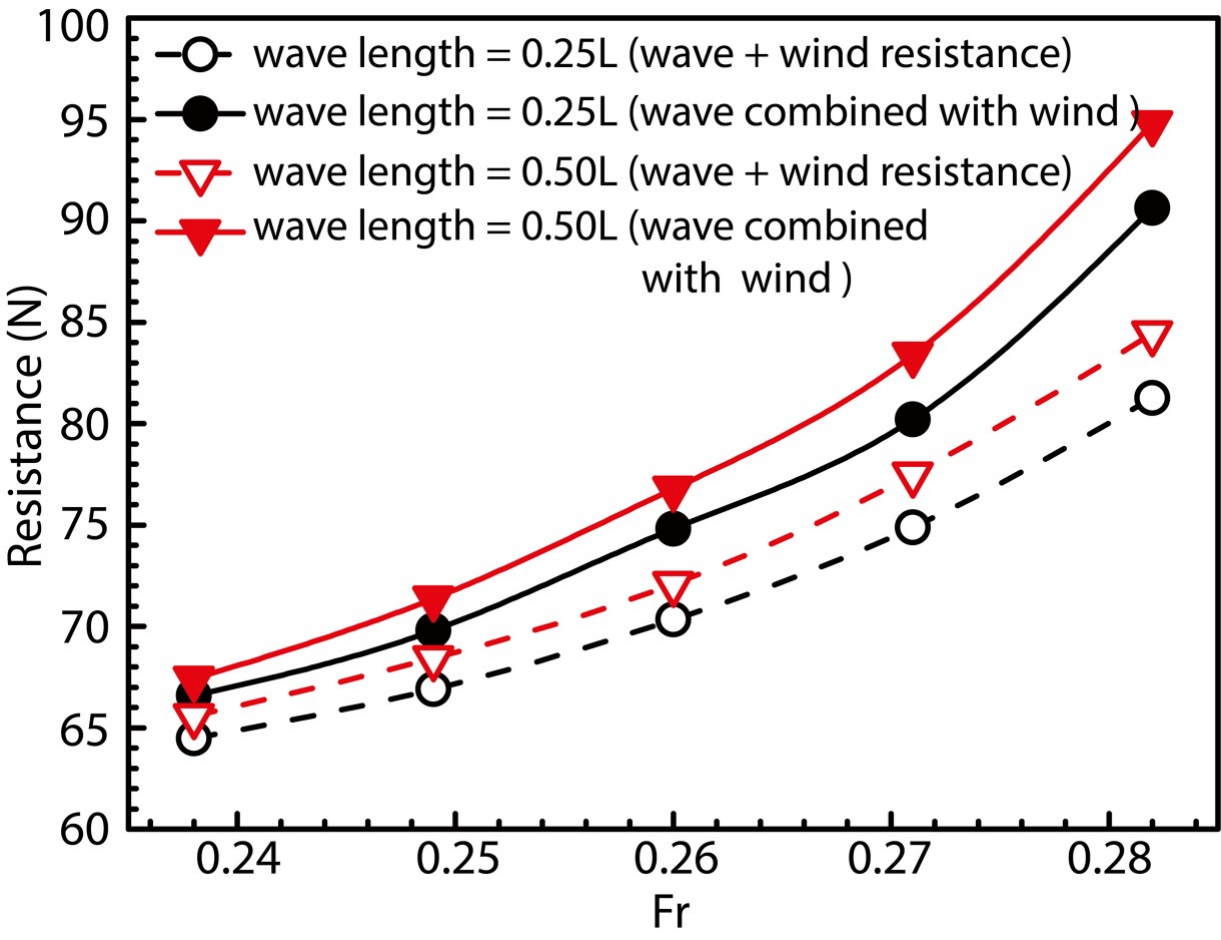
Comparison between resistance offered under combined wind–wave loads and that offered under isolated wave and wind loads.

To simulate the load of a ship subjected to combined wind and wave loads, a wind angle of 0° was selected to provide the wind load produced by oncoming first-order linear waves with wavelength ratios of *λ*/*L* = 0.25 and 0.50. Ship speeds considered during simulation were identical to those considered for the isolated wave-load case. Results obtained from these simulations are shown in Fig 16, wherein the solid and dotted lines represent the resistance due to combined wind–wave loads and the sum of individual wind and wave resistances, respectively. The colors black and red, represent cases corresponding to wavelengths of 0.25*L* and 0.50*L*, respectively. The purpose of Fig 16 is to compare the resistance to ship motion under combined wind and wave load against that observed under isolated wave- and wind-load conditions. The figure also demonstrates the effect of the combined action of wind- and wave-load on ship resistance. As observed, the resistance under conditions of combined wind–wave loads was greater compared to the sum of resistances due to individual wind and wave loads, and this enhancement in resistance increases with increase in ship speed. In fact, the resistance under the combined action of wind and wave is more than twice that under the action of wave alone.load Existence of such a large value of wind resistance seems unreasonable in comparison to the resistance caused by waves alone. During actual ship navigation, wind resistance for a general vessel typically equals about 2% of the total resistance. In the case of container ships, this value is usually high and may even reach up to 10%. Figs 13 and 16 show resistances observed under all conditions (calm-water, wave action, and wind action) at the model scale. Converting results shown in Figs 12 and 15 to the ship scale and considering the design speed to correspond to Fr = 0.26, we observed that when the wind speed inside the wind tunnel satisfies the critical Reynolds number criterion, the obtained wind-resistance coefficient does not change in value upon conversion to the ship scale. Wind resistance of the ship-scale superstructure was calculated in accordance with the wind-resistance formula, and the non-dimensional wind resistance coefficients formula [40]:

$C_{d}=\frac{F_{ds}}{\frac{1}{2}\rho V^{2}A_{s}}$, where *A*_*s*_ represents the front area in ship scale, *V* represents the mean wind speed and *ρ* is the density of air and *F*_*ds*_ represents the total wind resistance of the superstructure in the ship scale. Under actual navigation conditions, frontal area *A*_*s*_ of the full-scale ship model equaled 519.6 m^2^, air density *ρ* = 1.2047 kg/m^3^, C_d_ = 0.6725, and the mean wind speed equaled 20 m/s. Using these values, the total wind resistance of the full-scale superstructure equaled 84.192 kN. Ship resistance observed under the calm-water condition was also converted to its full-scale equivalent using the Froude method (two-dimensional method), and the resulting value equaled 1865.80 kN with the wind resistance amounting to nearly 4.51% of the total calm-water resistance of the full-scale ship.

**Fig 17:**
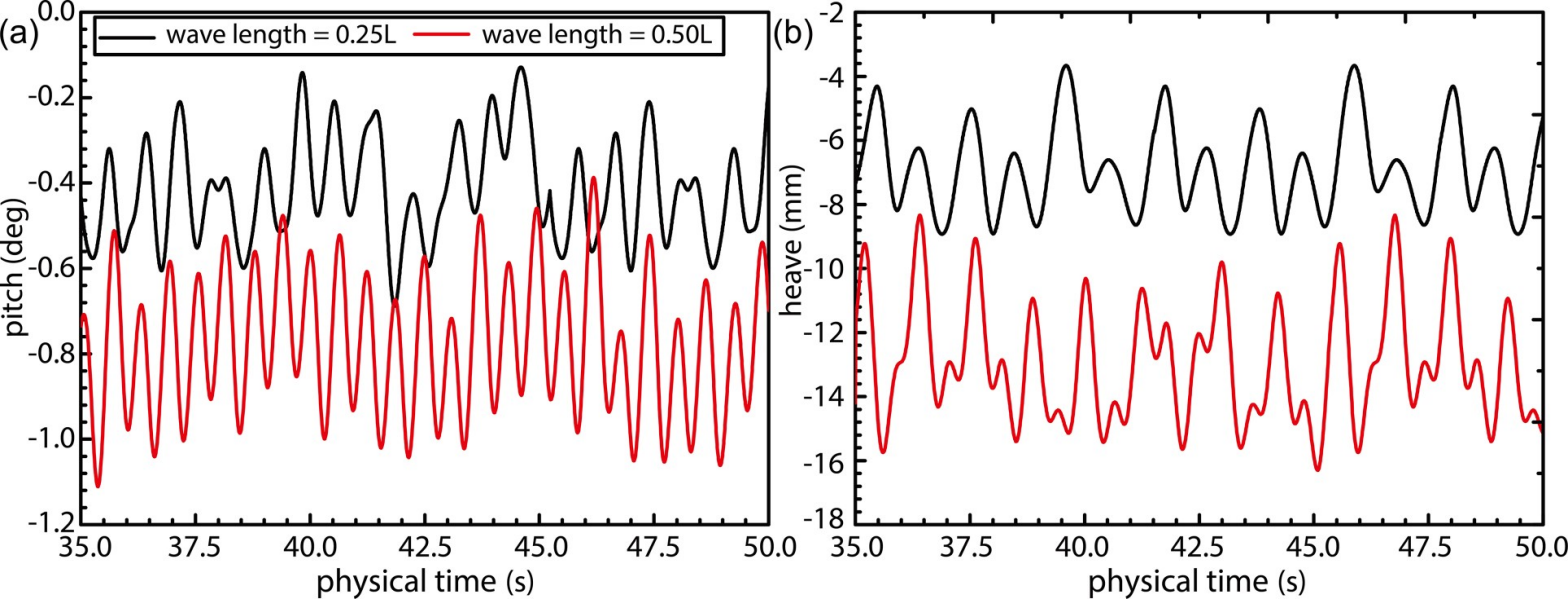
Results obtained for case involving combined wind–wave loads—(a) Time history of ship pitch; (b) Time history of ship heave.

**Fig 18:**
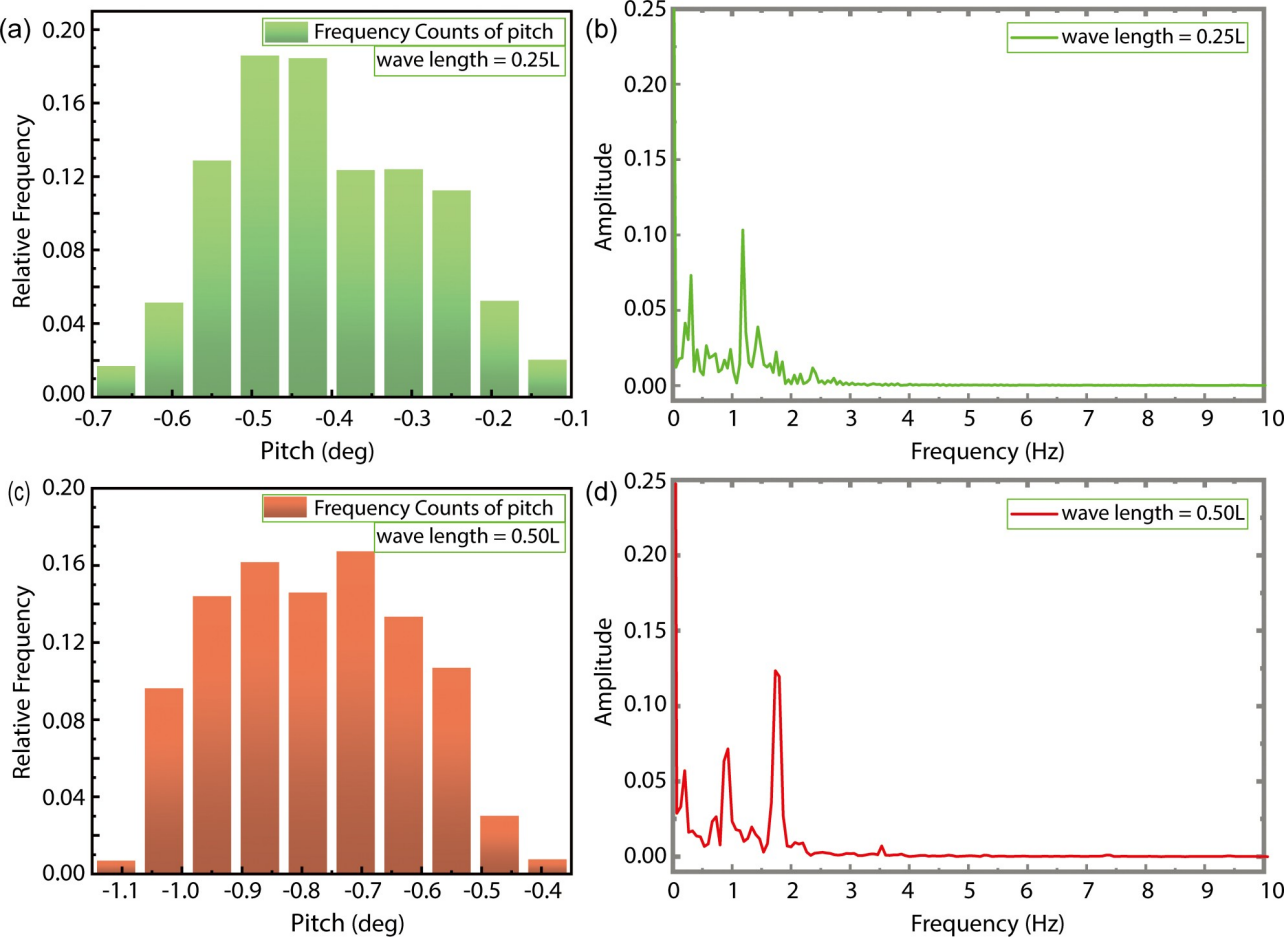
The relative frequency counter of pitch value (a. λ/L = 0.25; c. λ/L = 0.50), and the frequency domain curve after FFT of time history pitch (b. λ/L = 0.25; d. λ/L = 0.50).

**Fig 19:**
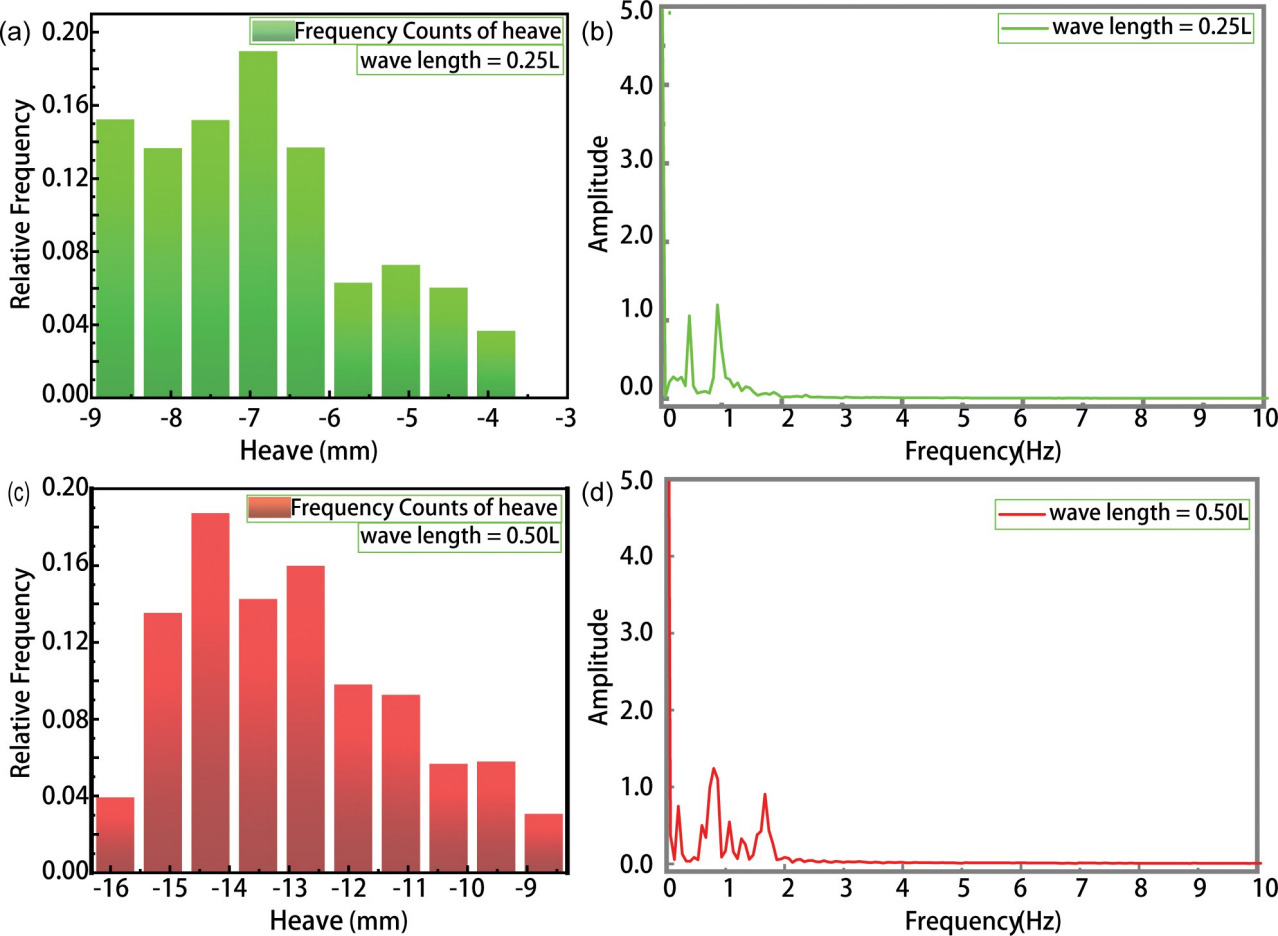
The relative frequency counter of heave value (a. λ/L = 0.25; c. λ/L = 0.50), and the frequency domain curve after FFT of time history heave value (b. λ/L = 0.25; d. λ/L = 0.50).

Fig 17 shows the time evolution of ship-motion characteristics in the presence of combined wind–wave loads. Figs 18 and 19 illustrates the relative frequency counter of pitch and heave value of Fig 17, and the frequency domain curve after FFT of time history pitch and heave value of Fig 17, respectively. Based on the pitch and heave curves shown in Fig 17 and relative frequency counter shown in Figs 18 and 19, it may be inferred that amplitudes of ship motion increase under combined wind–wave loads. Compared to the average head trim shown in Fig 14, significantly greater dipping of the bow can be observed in this case. The average draft also demonstrates an increase, which further increases the resistance to ship motion. Furthermore, effects of the fluctuating wind field on waves tend to alter wave parameters over the course of the voyage, thereby increasing the height and speed of waves.

**Fig 20:**
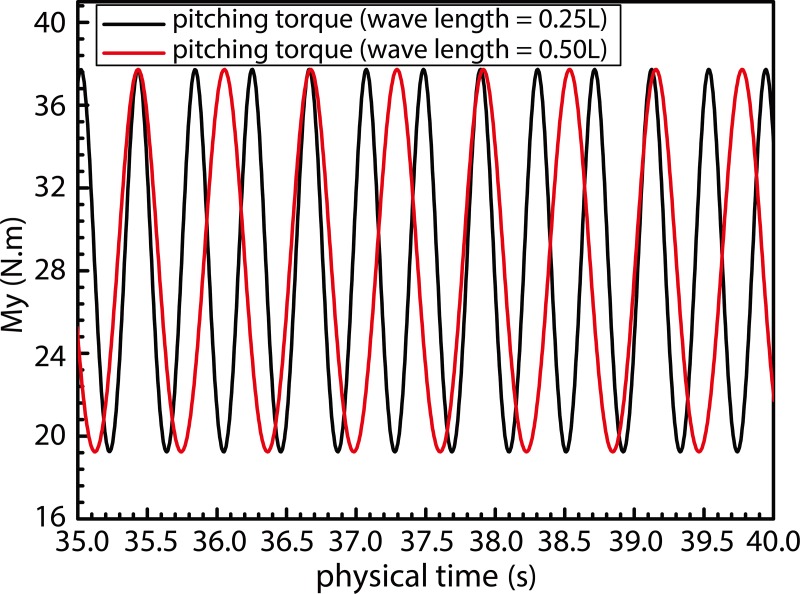
Pitching torque applied by fluctuating wind loads on the ship.

Fig 20 illustrates the time history of pitching torques acting on the ship under exclusive action of fluctuating winds under operating conditions defined by values of λ/L = 0.25 and 0.50. As observed, pitching torque of the fluctuating wind load acting on the ship is positively correlated with the square of the wind speed, and ship motions under the said wave loads exhibit the same frequency as that of the pitching torque. Therefore, motion responses of the ship resonate with the fluctuating wind field in the presence of combined wind–wave loads. This, in turn, increases the magnitude of the ship motion.

To investigate the effect of fluctuating wind fields on ship-motion resistance, motion resistance of the fixed ship model under combined wind and wave load was compared to that of a free model to analyze the overall resistance caused by the combined load. Draft and trim angles of the fixed model were set equal to those of the free model.

**Fig 21:**
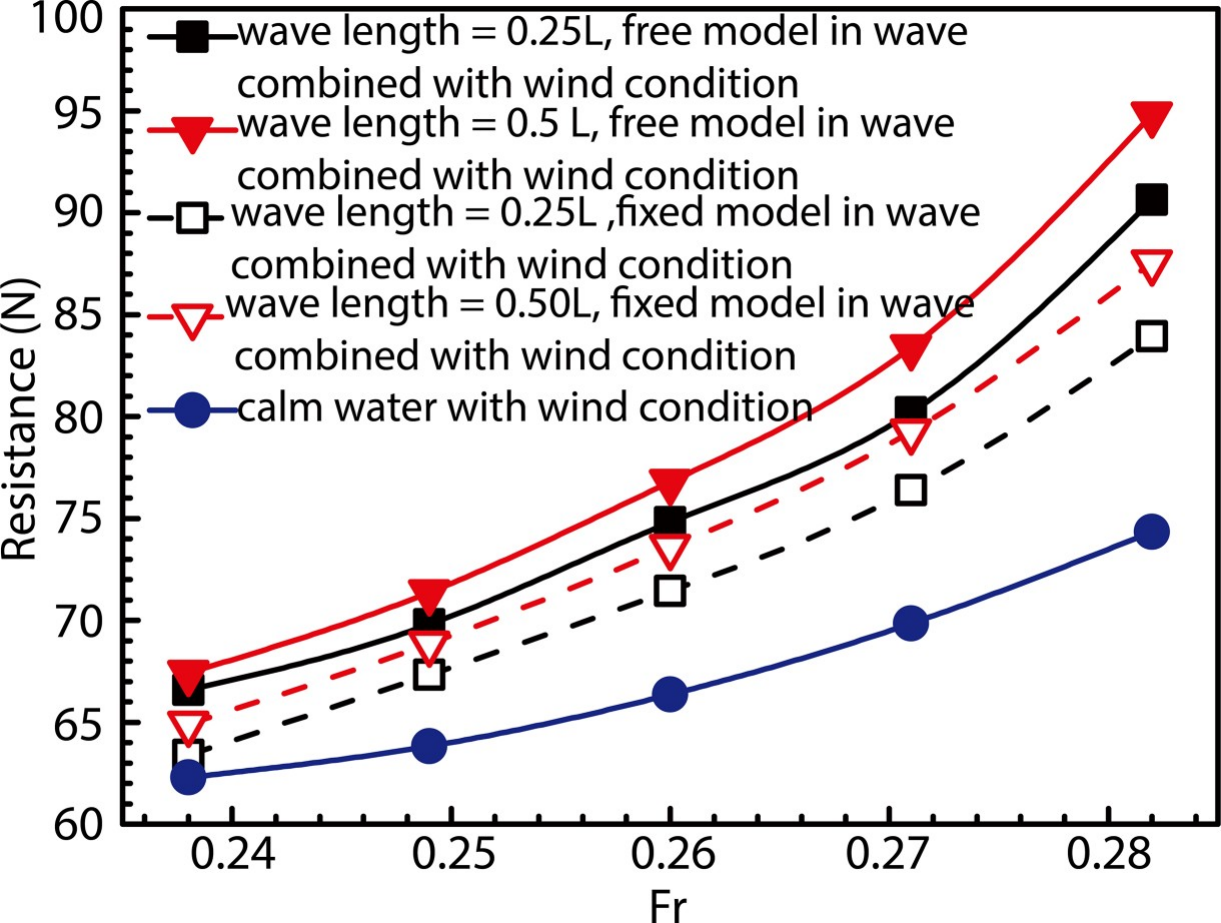
Comparison between ship resistance obtained using free and fixed models in presence of combined wind–wave loads.

**Fig 22:**
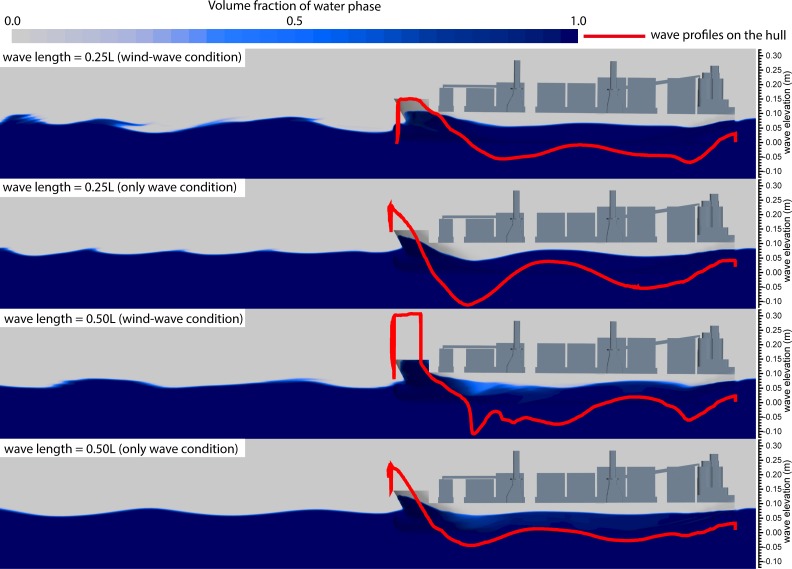
VOF phase diagrams and wave profiles on the hull corresponding to different wavelength cases.

**Fig 23:**
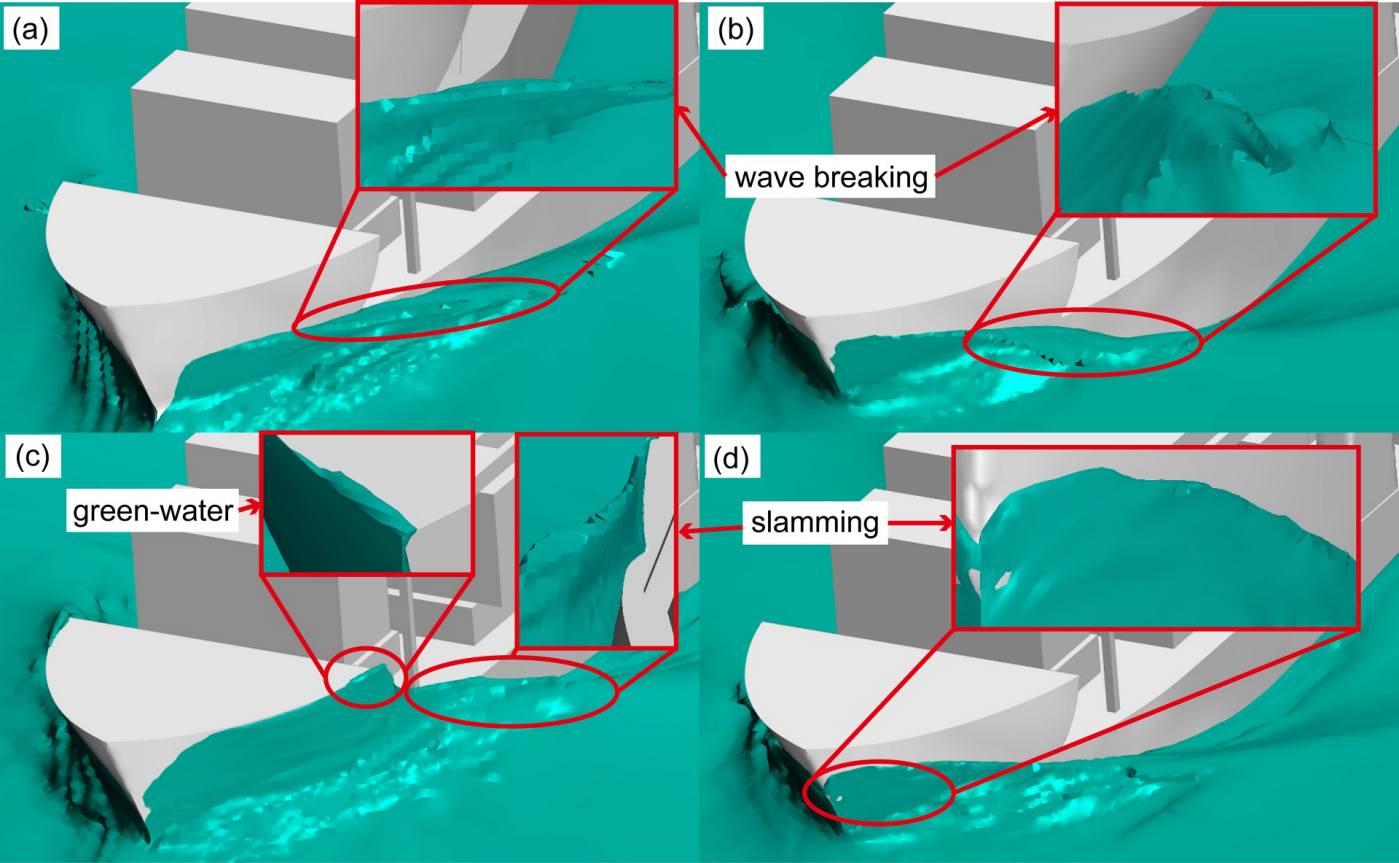
Ship and free surface interaction phenomenon diagrams corresponding to different wavelength cases.

Fig 21 shows resistance curves obtained for the fixed and free models subjected to the aforementioned combined load with wavelengths of 0.25*L* and 0.50*L*. In the figure, the solid curve with blue solid dots represents the algebraic sum of calm-water- and wind-load-induced resistances. The sum of resistances (denoted by solid red and black curves) in the free-model case under combined wind and wave load was considered as the target total resistance, whereas that of resistances (denoted by red and black dotted lines) in the fixed-model case under identical load conditions was considered as the target total resistance upon removal of the influence of model movement. The algebraic sum of calm-water and wind resistances was considered the total model resistance after removal of the influence of model motion, wave resistance, and resistance offered by the coupled wind–wave effect, which is known from the difference in the above resistance. The effect of the wave added resistance and the added resistance under the wind-wave coupling effect is greater than the resistance of the model motion. Fig 22 shows the VOF distribution of water and air components under combined wind and wave load at wavelengths of 0.25 *L* and 0.50 *L*. Fig 23 shows the ship and free surface interaction phenomenon diagrams corresponding to different wavelength cases. As observed, the fluctuating wind field causes the wavelength and height of waves to change irregularly at a wavelength-to-ship length ratio given by *λ*/*L* = 0.25. Usually, the breaking of waves must lead to formation of vorticity and loss of kinetic energy; however, in the present case, the wave did not break automatically. Instead, it was broken by fluctuating winds. The presence of fluctuating wind increases wave velocity at the crest, which in turn, increases the wave kinetic energy. Additionally, fluctuating winds destroy the original wave shape, thereby altering wave parameters. The wave condition with λ/L = 0.50, wherein the wavelength increases without any change in wave height, was observed to be characterized by a wave steepness smaller than that of the wave with λ/L = 0.25. Therefore, the wave condition with λ/L = 0.50 could be considered to possess a low surface-roughness level along the wave surface when compared against the wave condition with λ/L = 0.25. Therefore, the effects of wind field on the waveform were less pronounced under wave conditions with λ/L = 0.50. In the case with *λ*/*L* = 0.25, crest breaking was observed to occur during wave propagation under the action of the wind field. This subsequently led to the formation of plunging waves. In comparison, in the case with *λ*/*L* = 0.5, the occurrence of crest breaking was observed to be less pronounced, albeit with severe shipping of water onto the bow and increased impact of waves on the ship bow. Considering the results shown in Fig 14(B), it may be inferred that waves are rapidly "lifted" by the wind prior to breaking at the crest. Subsequently, the said waves rapidly "fall" after separating from the wave surface. Under the influence of the wind field, waves undergo several cycles of fluctuation before encountering ship motion, and the resulting increase in wave kinetic energy enhances the wave pressure against the ship bow. In addition, the diffraction of waves leads to formation of a diffraction force.

**Fig 24:**
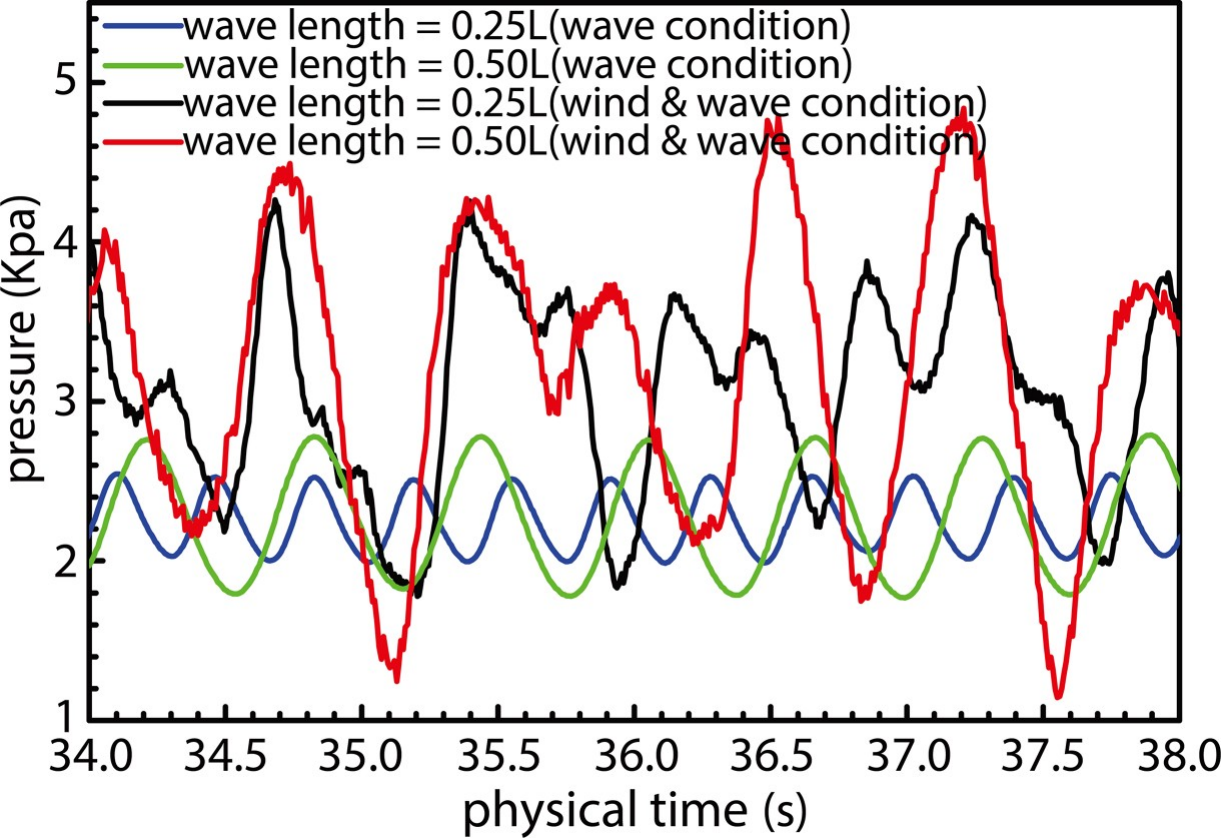
Pressure curves at ship bow under different load conditions.

**Fig 25:**
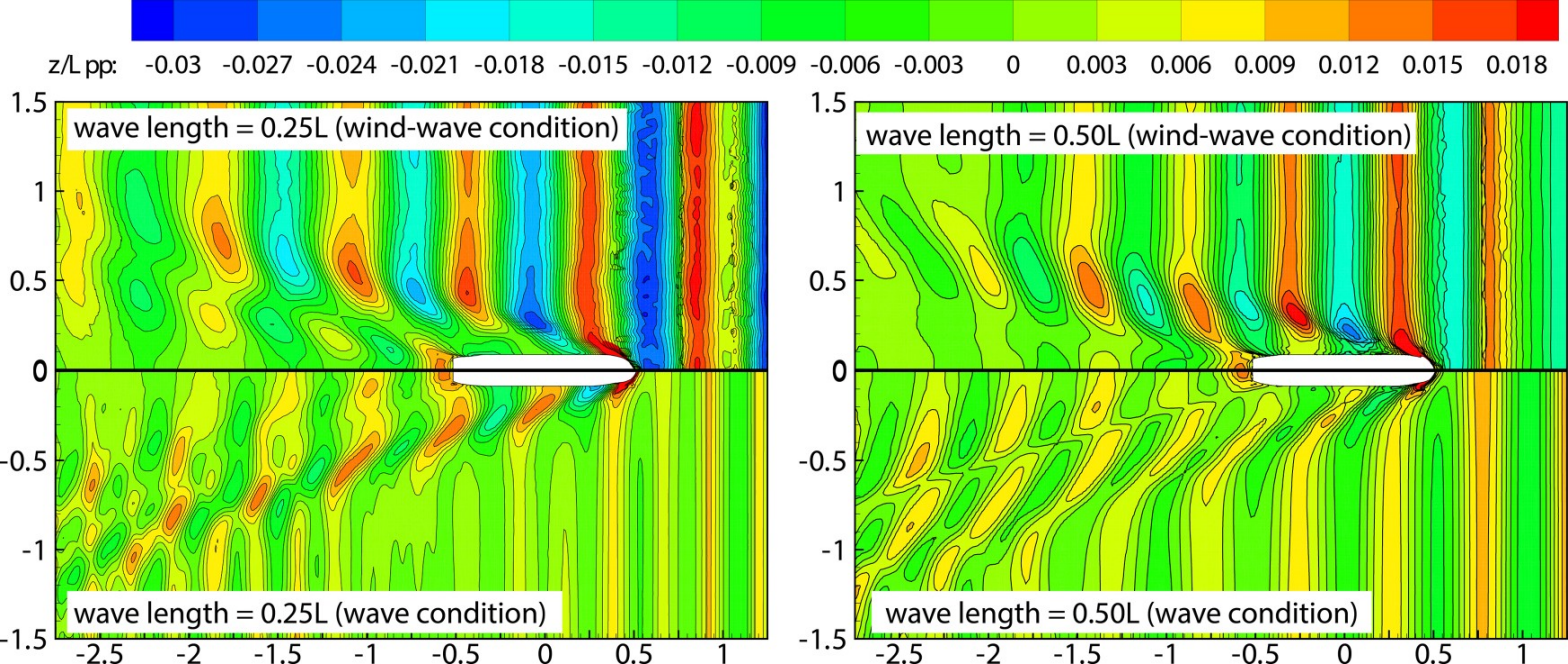
Wave elevation contour maps under different operating conditions. Left: *λ*/*L* = 0.25; Right: *λ*/*L* = 0.50.

Fig 24 illustrates curves corresponding to pressures acting on the surface of the ship bow at depth 0.5 *H* below the free surface under influence of the combined wind-and-wave and isolated wave load conditions. Additionally, Fig 25 illustrates the wave-surface contours obtained under different operating conditions. A comparison of the bow-pressure curves obtained for the said two loads under conditions of *λ*/*L* = 0.25 and 0.50 reveals that an increased pressure acts on the bow under combined wind–wave loads. This effect manifests in the form of increased average-pressure and pressure-fluctuation magnitudes. Pressure curves corresponding to combined wind–wave loads exhibit a certain degree of irregularity owing to changes in regular wave parameters caused during wave propagation owing to the influence of fluctuating wind fields. The observed impact of waves on the ship bow was also irregular. As can be observed from pressure curves obtained for combined wind–wave load cases with wavelengths of 0.25*L* and 0.50*L*, breaking waves are formed and developed by the action of fluctuating wind fields until they eventually encounter the ship. A "double humped" pressure curve can be observed in the case with wavelength of 0.25*L*. Given that the waves, in this case, are rather steep, their shapes are unstable during propagation owing to effects of the wind field. This results in overlapping of adjacent crests to form a large "double humped" peak, as shown in Fig 16(A). When such waves encounter the ship bow, the wave surface becomes elevated and corresponding wave pressure begins to increase as the bow encounters the wave crest for the first time. The observed wave pressure subsequently declines after reaching the first extremum of the "double humped" peak, and the second extremum occurs upon completion of a similar cycle of events. The pressure curve corresponding to the case with wavelength equal to 0.50*L* has only a single peak because waves, in this case, are less steep and do not break completely, thereby becoming more stable. Therefore, the first wave–ship encounter lasts longer, and the wave surface "climbs" higher up the bow whilst causing an increase in wave pressure. In Fig 16(B), this effect has been shown as an increase in the green water level on the bow of the ship.

Upper and lower halves of Fig 25 illustrate wave-height curves corresponding to the combined wind–wave and isolated wave load conditions, respectively. As observed, the waves clearly exhibit diffractive effects when they encounter the ship. The wave height at the bow is greater under combined wind–wave load, and the density of the wave-height curves indicates that the corresponding gradient is steeper in this case. When a wave encounters the ship, the incident wave energy is affected upon the its impact with the ship, and the viscous effect of the hull along with the ship–wave interaction (except the viscosity effect) results in diffraction of waves, thereby causing a diffraction force to act on the ship. Wave dissipation outside the Kelvin-waveform range is caused by significant changes in wave shape due to repeated wind and wave interactions. As shown in Fig 14(B), the waveform, in this case, is characterized by sharp peaks and rounded valleys, and that such a shape cannot be maintained for long-distance ships, because breakage and dissipation occur during propagation. With regard to wave dissipation within the Kelvin-waveform range, the waveform difference can be obtained at the end of the waveform region, except for dissipation of the wave itself (same dissipation as that of the Kelvin waveform). The remaining wave is dissipated, thereby resulting in increased resistance to ship motion upon absorption of wave energy. As can be realized from the analysis of ship motion shown in Figs 14 and 17 and ship resistance in Figs 16 and 20, the diffused portion of waves corresponds to wave resistance, additional resistance observed under combined wind–wave load, and resistance offered owing to ship motion. Compared to individual wave conditions, the increased wave dissipation has a greater contribution to the resistance encountered by the ship under combined wind–wave load. This implies that wave diffraction in the presence of combined wind–wave load results in increased dispersion of wave energy and strength of diffractive forces acting against ship motion.

## 5. Conclusions

This study investigates the additional resistance encountered by a ship subjected to combined wind and wave load by performing a series of numerical calculations and experimental measurements. Results obtained for the combined load case were compared against those obtained for cases involving action of isolated wind and wave loads. Following conclusions can be drawn from this research.

1. Results obtained via numerical calculations and experiments are broadly consistent, thereby demonstrating high accuracy. This indicates that the meshing, fine-mesh processing, and turbulence models used for the isolated wind- and wave-load calculations satisfy the prerequisites for computational accuracy. Use of an overset mesh for estimating the effect of combined wind–wave loads (after additional fine processing) is a viable option.
2. The additional resistance encountered by the ship under combined wind–wave load exceeded the algebraic sum of the resistances encountered under isolated wind and wave loads. This is attributable to the following factors—(i) under combined wind–wave load, the fluctuating wind field applies an unsteady pitching torque on the ship, thereby increasing the pitch and heave motions as well as motion resistance; (ii) generated waves are affected by the wind field during propagation; this causes a change in parameters, such as wave height and wave speed, thereby increasing impact pressures acting on the bow. (iii) wave diffraction in the combined-load case is more intense, thereby resulting in stronger dispersion of wave energies at the stern as the incoming wave gets diffracted by the ship. This results in greater wave-height attenuation around the stern (compared to the isolated wave load condition) and increased diffractive forces acting on the ship.
3. Wave conditions considered in this study were exclusively based on short waves. Fixed-model calculations under combined load conditions were found to yield slightly lower wind and wave resistance at each ship speed compared to the free model. Therefore, motion resistance accounts for only a small portion of the increase in resistance associated with the action of combined wind–wave loads. The observed increase in resistance is largely caused by wave parameter alterations—owing to influence of the wind field on short waves (which increases the difference in pressure between the bow and stern)—and increased diffractive forces acting on the ship. Therefore, bow-shape optimization or the use of bulbous bows could help reduce the added resistance faced by ships operating under actual oceanic conditions.
4. A lack of experimental data concerning added resistance encountered by ships under combined wind and wave loads meant it was not possible to validate the computational accuracy of added-resistance values obtained. Nonetheless, the results obtained in this study provide a reference for calculating the added resistance when sailing through actual wind–wave load conditions. Based on the findings of this study, the authors intend to focus future research endeavors on ship resistance and self-propulsion under combined wind–wave load conditions. The objective then would be to deduce equations that convert the resistance of ship models under these conditions to those of actual ships, thereby providing a reference for calculation of ship resistance under actual oceanic conditions.

### 5.1 Limitations of research and future recommendations

Although wind-load coefficients of superstructure containers set at different wind angles under full-load conditions, wave resistances encountered by superstructure-less models under a variety of wave conditions, and wind–wave resistance of the full-scale models in the presence of waves coupled with a fluctuating wind field have been investigated in this study, there still exist certain limitations. First, given that wave steepness and wavelength-to-ship-length ratios were different for the cases considered in this study, understanding the influence of wave parameters on obtained results was difficult. Second, all investigations were performed at the model scale, and the coupling effect between wind and waves at different scales, including the ship scale, was not considered. Future research recommendations include (i) consideration of cases involving waves with the same wavelength and different wave steepness as well as those with the same wave steepness and different wavelengths; (ii) comparative study between CFD, experiments, and linear potential theory; (iii) research concerning the influence of coupled wind–wave load under at different model scales.
